# Ecological membrane for slope engineering based on red bed soil

**DOI:** 10.1371/journal.pone.0286949

**Published:** 2023-06-08

**Authors:** Haoqiang Lai, Cuiying Zhou, Zhen Liu

**Affiliations:** 1 School of Civil Engineering, Sun Yat-sen University, Guangzhou, P. R. China; 2 Guangdong Engineering Research Center for Major Infrastructures Safety, Guangzhou, P. R. China; Middle East Technical University, TURKEY

## Abstract

Ecological slope protection projects (such as the reinforcement of low slopes by plants and ecological restorations of the soil of high steep rocky slopes) are essential for restoring the natural environment. In this study, red bed soil and composite polymer adhesive materials were used to develop an ecological membrane for application in slope ecological protection. The basic physical and mechanical properties of the ecological membranes with different material percentages were studied through tensile strength test and viscosity test, the effect of different material percentages on the properties of ecological membranes was studied, and the soil protection performance and ecological restoration performance were studied through anti-erosion and plant growth tests. The results show that the ecological membrane is soft and tough, with high tensile strength. The addition of the red bed soil can enhance the strength of the ecological membrane, and the ecological membrane with 30% red bed soil has the highest tensile strength. The ecological membrane has considerable tensile deformation capability and viscosity, and up to 100% by mass, the more composite polymer adhesive materials added, the greater the tensile deformation capability and viscosity. And the ecological membrane can enhance the anti-erosion performance of the soil. This study clarifies the development and technology of the ecological membrane, reveals the effect of different material percentages on the properties of ecological membrane, and analyzes the slope ecological protection mechanism of the ecological membrane, thereby providing theoretical and data support for its development, improvement, and application.

## 1 Introduction

Slope engineering might cause hazard to the environment, such as damaging vegetation and causing landslides, researchers have conducted many studies on slope protection in the pursuit of slope environment protection and/or restoration. At present, the methods for slope protection mainly include engineering measures and plant restoration measures [1, 2]. Engineering measures such as shotcrete, lattice beams, and retaining walls can achieve rapid and effective slope protection and provide safety and stability. However, such methods cannot restore the natural environment of the slope. The slope protection ultimately achieved is not harmonious with or friendly to the natural environment and can lead to the release of many materials harmful to the natural environment. Thus, it fails to truly achieve land protection, soil health, and geo-ecological restoration [3]. Planting measures are mainly used to reduce evaporation and erosion of soil by ecological restoration, such as plant restoration. These protect the slope and restore the natural landscape by enhancing the soil consolidation and water retention capacity of the plants. At present, the more promising research field in slope protection is that of planting measures, as these meet the current demands of human society with regard to environmental protection [4].

An "ecological blanket" is a means of slope protection through planting measures. It is an effective slope protection method that uses plant restoration and is commonly used across nations. It comprises covering the soil surface with a reinforced mesh or fiber layer to protect the slope soil and reduce its erosion loss while adding grass seed, water retaining agents, nutrient soils, and other materials to restore the environment of the slope. The protection of the ecological blanket can reduce the slope erosion by wind, heating cooling and setting drying cycles while providing physical protection. After the plants are fully grown, the soil plants are restored, and long-term slope protection can be achieved [5, 6]. Research on the slope protection of the ecological blanket mainly involves the selection of blanket fabric and fibers and that of plants. The slope protection performance and mechanisms are mainly studied through anti-erosion and plant growth tests. The ecological blanket has been used in various slope protection projects, such as in slope ecological restoration, riparian engineering protection, and rock slope protection [7–10]. However, the ecological blanket has certain limitations. When the plants in the ecological blanket are not yet fully grown, the ecological blanket plants can disperse and slow down rainwater runoff to reduce erosion, but this also indicates that the slope protection performance of the ecological blanket at the initial stage under rainstorm conditions is somewhat limited. Also, the slope surface must be leveled before the application of the ecological blanket, rendering it difficult to achieve a good ecological restoration effect for undulating soils and high and steep slopes. Cuiying (Sun Yat-sen University, China) studied the application of green and environment-friendly composite polymer adhesive materials in the ecological restorations of rocky slopes and found that these materials exhibited drying and membrane-forming characteristics [11, 12]. An ecological membrane prepared with a composite polymer adhesive material is viscous, and covering the ecological membrane over the soil surface can the protect the soil and conserve soil water and moisture, which is conducive to plant growth. Thus, the ecological membrane can be used in slope protection and ecological restoration. However, the tensile strength of the ecological membrane material is not sufficient, leading to great limitations in its applications in ecological slope restoration. In this study, red bed soil with good hydrophilicity and stickiness [13, 14] and a composite polymer adhesive material with a dry membrane-forming property are used to develop an ecological membrane. Red bed mainly refers to a set of continental and shallow lake sediments with red mudstone, siltstone, sandstone and other lithology. Red bed weathered soil has high clay mineral content and strong hydrophilicity. By mixing the red bed soil into composite polymer, we can produce the ecological membranes with significantly improved tensile strength, making it conducive for construction and applicable to various slope protection projects, such as those in steep slopes.

To develop a new type of high-performance, green, and environment-friendly slope protection material and method, this study investigated the development of an ecological membrane for slope engineering based on red bed soil. By developing a high-performance ecological membrane with a high tensile strength, the ecological protection of the slope can be better realized. The membrane can also play an important role in slope protection in certain extreme conditions, such as steep slopes. This study analyzed the development method and technical details of the ecological membrane and examined the performance of ecological membranes made of different materials in terms of tensile strength and viscosity. It also analyzed the membrane-forming principle of the ecological membrane and optimized its development. Moreover, this study clarified the performance and effect of the ecological membrane in slope ecological protection through anti-erosion and plant growth tests.

## 2 Materials and method

A composite polymer adhesive material is a type of water-based nano adhesive with drying and membrane-forming characteristics, which acts as the foundation for the development of the ecological membrane. However, the strength of an ecological membrane developed only with an adhesive is low, which is not conducive to its application in slope ecological restoration. The aim is to develop an ecological membrane with high tensile strength and toughness by mixing composite polymer adhesive materials with red bed soil. The tensile strength of the ecological membrane is high, and it exhibits good performance in slope protection. The influences of the addition of different red bed soil on the performance of the ecological membrane were studied through tensile strength and viscosity tests. Furthermore, the effects and mechanisms of the slope protection and ecological restoration of the ecological membrane were studied through anti-erosion and plant growth tests.

### 2.1 Development of the ecological membrane

#### 2.1.1 Materials

The raw materials of the ecological membrane include the red bed soil common in South China and composite polymer bonding materials. Red beds are widely distributed in China and the world [15–19]. The red bed soil used in this study was procured from the common red bed mudstone found in South China. This reduced the cost of the ecological membrane and made the process environment friendly. The composition and characteristics of red bed soft rock are generally characterized by a high clay mineral content, strong hydrophilicity, and hard soil particles. The content of clay minerals in the red bed soil is high; thus, it has strong hydrophilicity and good viscosity. As shown in Table 1, the Empyrean X-ray powder diffractometer was used to determine the content of red bed soil, the quartz content in the weathered soil sample of the red bed is the highest at 74.13%, followed by kaolinite, illite, chlorite, and montmorillonite, accounting for 19.06%. It can be inferred that the weathered soil of the red bed is cohesive and hard [20].

**Table 1:** Mineral content of red bed soil.

Mineral	Content (%)
Quartz	74.13%
Illite	9.56%
Kaolinite	5.29%
Chlorite	3.04%
Montmorillonite	1.18%
Anorthite	2.74%
Hematite	4.08%

**Fig 1:**
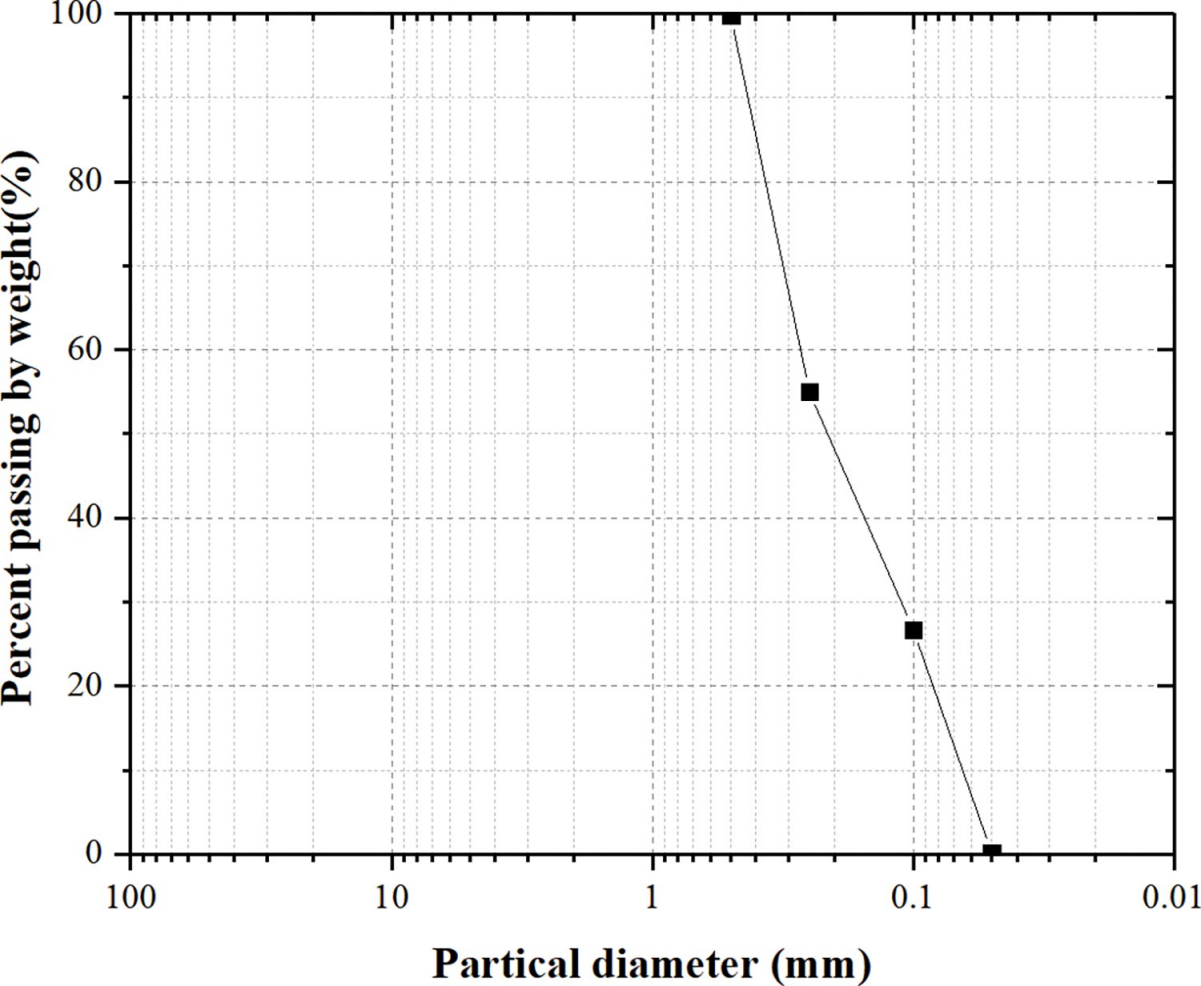
The particle size distribution of the red bed soil.

According to the laboratory tests on the red bed soil, the particle size of the red bed soil was shown in Fig 1, and the atterberg limits and Gs of the red bed soil were shown in Table 2.

**Table 2:** Properties of red bed soil.

Soil properties	Plastic limit	Liquid limit	Dry density	hygroscopic water content
Value	36.5%	55.2%	1.24g/cm^3^	3.7%

The composite polymer adhesive material is an organic material resistant to hydrolysis. It is a white viscous lotion and insoluble in water at room temperature. However, the material has good dispersibility in water and can be compounded with water to form a dispersion solution. The effective polymer substances of the composite polymer adhesive materials exist in the form of latex particles with strong adhesion in the aqueous dispersion. The composite polymer adhesive material has good stability at room temperature. The adhesive material also displays a membrane-forming property after drying and can form an elastic and tough membrane, which forms the foundation for developing an ecological membrane. The degradation cycle of the composite polymer adhesive material is 2 years, and the degradation products are CO_2_ and H_2_O, which do not generate any substances that are toxic and/or harmful to the environment. Thus, the adhesive material is an ecological material [21, 22].

#### 2.1.2 Development process of ecological membrane

As mentioned above, the red bed soil was obtained from the red bed soft rock in South China. The red bed soil samples had a high clay content, and good water retention and were not easily dispersed in water. Each soil sample was air dried and then crushed by a machine. After passing a standard laboratory sieve, it was stored in a material tray for use. Subsequently, 100 g of water was added to the red bed soil and composite polymer bonding material in a beaker according to certain proportions, as presented in Table 3. The materials were fully mixed for 15 min with the STSJB-1000 laboratory pilot mixer, and then, the beaker was sealed with plastic wrap for use. A film casting method was used to prepare the ecological membrane. The materials prepared in the beaker were poured onto a transparent and smooth PVC (Polyvinyl Chloride) plate with a length, width, and thickness of 50 cm, 50 cm, 4 mm, respectively. The PVC plate was tilted until the materials were evenly spread on the plate to form a membrane with a thickness of approximately 0.2 mm (the thickness of the membrane can be adjusted with a scraper). The membrane was placed into a laboratory hot air circulation oven of constant temperature and dried at 60°C for 5 h to form the ecological membrane. The molded ecological membrane was peeled from the PVC plate (with a smooth surface) and was then packed in sealed bags for standby to prevent its physical characteristics from being affected by long-term exposure to air.

**Table 3:** Material addition of ecological membrane development.

Sample	Red bed soil (g)	Composite polymer adhesive materials (g)	Proportion of red bed soil (%)
Sample CK	0	230	0
Sample 1	23	207	10
Sample 2	46	184	20
Sample 3	69	161	30
Sample 4	92	138	40
Sample 5	115	115	50

**Fig 2:**
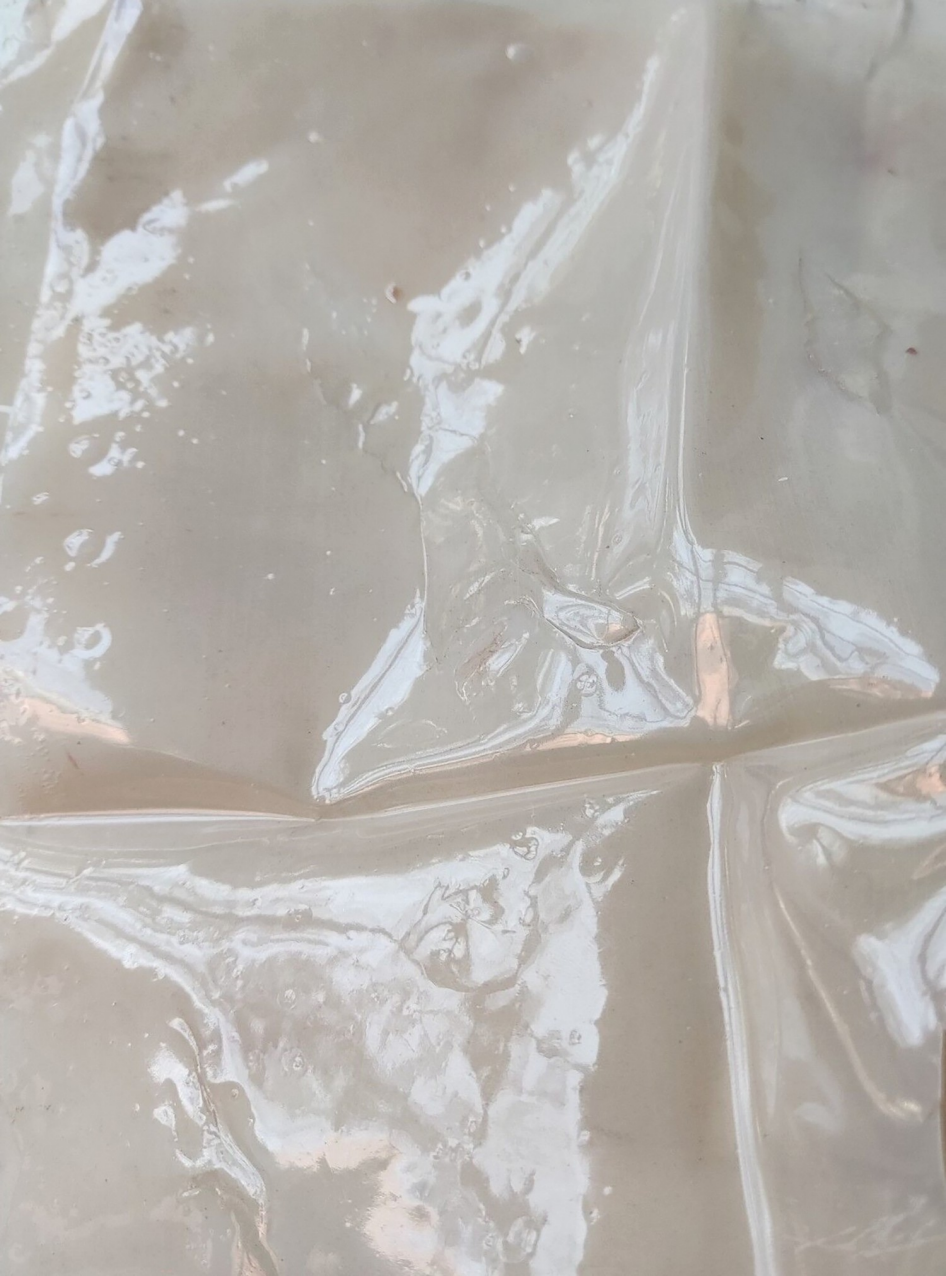
Ecological membrane without red bed soil.

**Fig 3:**
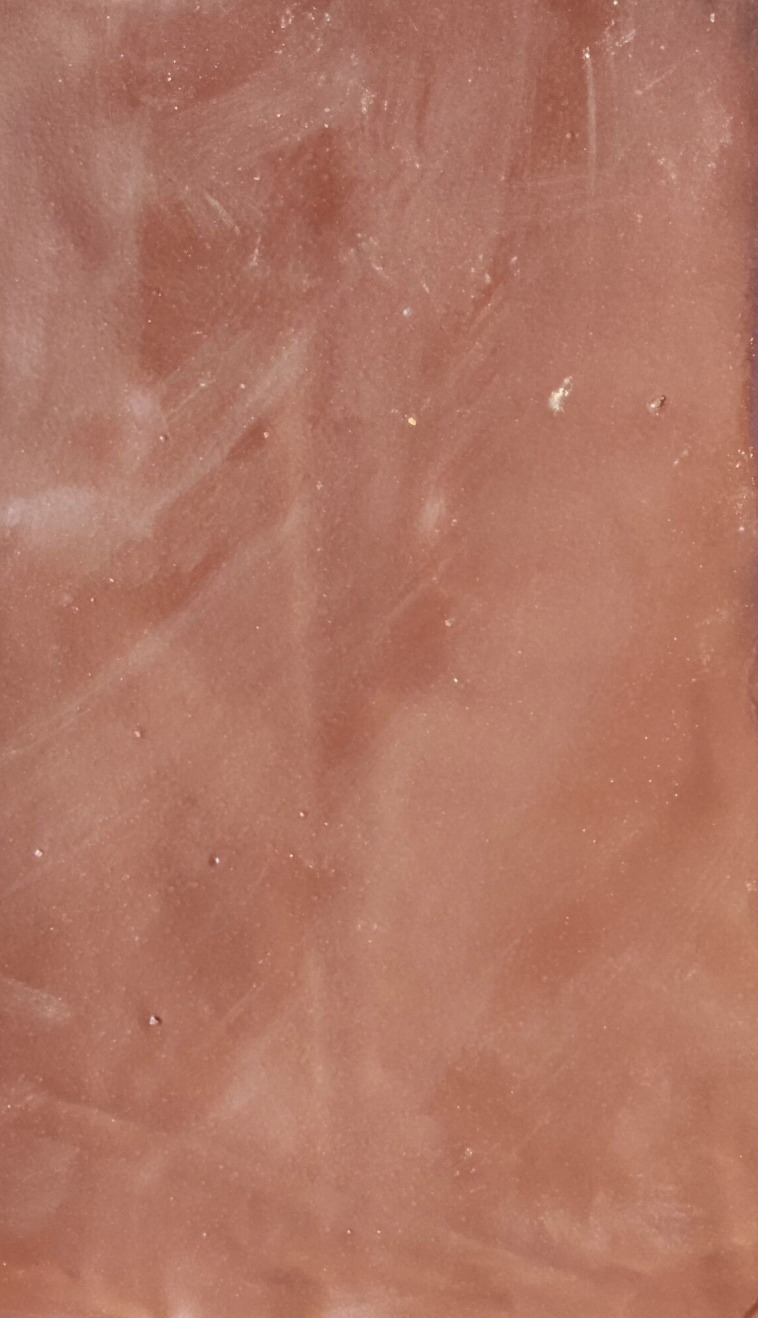
Ecological membrane added with red bed soil.

The ecological membrane was developed according to six different proportions of materials, as shown in Table 3. It was then packaged properly for use, and further relevant tests were conducted with the ecological membrane to study its characteristics to select the optimal material proportion for the ecological membrane. Figs 2 and 3 shows the developed ecological membrane. The ecological membrane without the red bed soil was milky white and that with the red bed soil was red, the same color as the red bed soil. The developed ecological membrane was soft, flexible, and foldable, with a thickness of approximately 0.2 mm (approximately equivalent to the thickness of two 4A printing papers).

### 2.2 Performance test of ecological membrane

The ecological membrane can cover the soil surface to protect the soil from erosion through its own viscosity and strength. In this study, the physical and mechanical properties of the ecological membrane, such as its tensile strength, toughness, and viscosity, were studied through mechanical tests. The effects of different material ratios on the mechanical properties of the ecological membrane were analyzed, and an anti-erosion test was conducted to study the slope protection and ecological performance of the ecological membrane.

#### 2.2.1 Tensile strength test

A strong tensile strength is the key to realizing the slope protection of an ecological membrane. In this test, six types of ecological membranes developed according to six proportions (Table 3) were used for tensile testing, and the tensile and deformation resistances of the ecological membranes with different material ratios were tested. Each sample underwent three parallel tests, with the average value being considered the final result. The specifications of the samples are listed in Table 4.

**Table 4:** Tensile strength test parameters.

Sample	Length (mm)	Width (mm)	Gauge length (mm)	Thickness (mm)	Mass (g)
Sample CK	120	30	100	0.2	3.6
Sample 1	120	30	100	0.2	3.6
Sample 2	120	30	100	0.2	3.7
Sample 3	120	30	100	0.2	3.9
Sample 4	120	30	100	0.2	3.9
Sample 5	120	30	100	0.2	4.1

The YH-LLA-200KG universal material tensile testing machine was used for tensile strength testing of the ecological membrane. Currently, there is no mechanical test standard for ecological membranes. This tensile test was conducted according to the standard test method for tensile and deformation resistance of plastics based on American Society for Testing and Materials (ASTM) D638-2003. The size and dimension of the tested samples was shown in Table 4. Before the test, pre-stretching was conducted within the elastic range of a small elastic deformation. According to the ASTM D638-2003 plastic tensile deformation resistance standard, a tensile rate of 50 mm/min was adopted for the test. The ambient temperature of this test was 20°C, and the relative humidity was 59%.

The calculations for the stress *σ* and strain *ε* of the ecological membrane sample in the tensile process are as follows.


$$\sigma =\frac{F}{A}$$ (1)

$$\varepsilon =\frac{L-L_{0}}{L_{0}}$$ (2)

In the above equations, *F* is the load applied to the samples during tension (N), *A* is the cross-sectional area of the ecological membrane sample before stretching (mm^2^), *L*_*0*_ is the gauge length of the ecological membrane sample before stretching (mm), and *L* is the length of the ecological membrane sample after tensile deformation (mm).

The Young’s modulus of elasticity *E* of the sample indicates the rigidity of the material; *σ*_*b*_ is the breaking strength or tensile strength of the samples, representing the maximum stress value that the sample can withstand before tensile failure. The calculation formulas are as follows.


$$E=\frac{F_{e}L}{A∙(L-L_{0})}$$ (3)

$$\sigma b=\frac{F_{b}}{b∙d}$$ (4)

$$W=\frac{F_{b}}{b∙L}$$ (5)

Here, *E* is the Young’s modulus of elasticity (kPa), *F*_*e*_ is the maximum tensile load at the elastic deformation stage (N), *σ*_*b*_ is the tensile or breaking strength (N/mm^2^), *F*_*b*_ is the maximum tensile load in the plastic deformation stage (N), *b* is the width of the sample (mm), *d* is the thickness of the sample (mm), and *W* is the shear strength (of material under zero normal stress)of the material.

#### 2.2.2 Viscosity test

**Fig 4:**
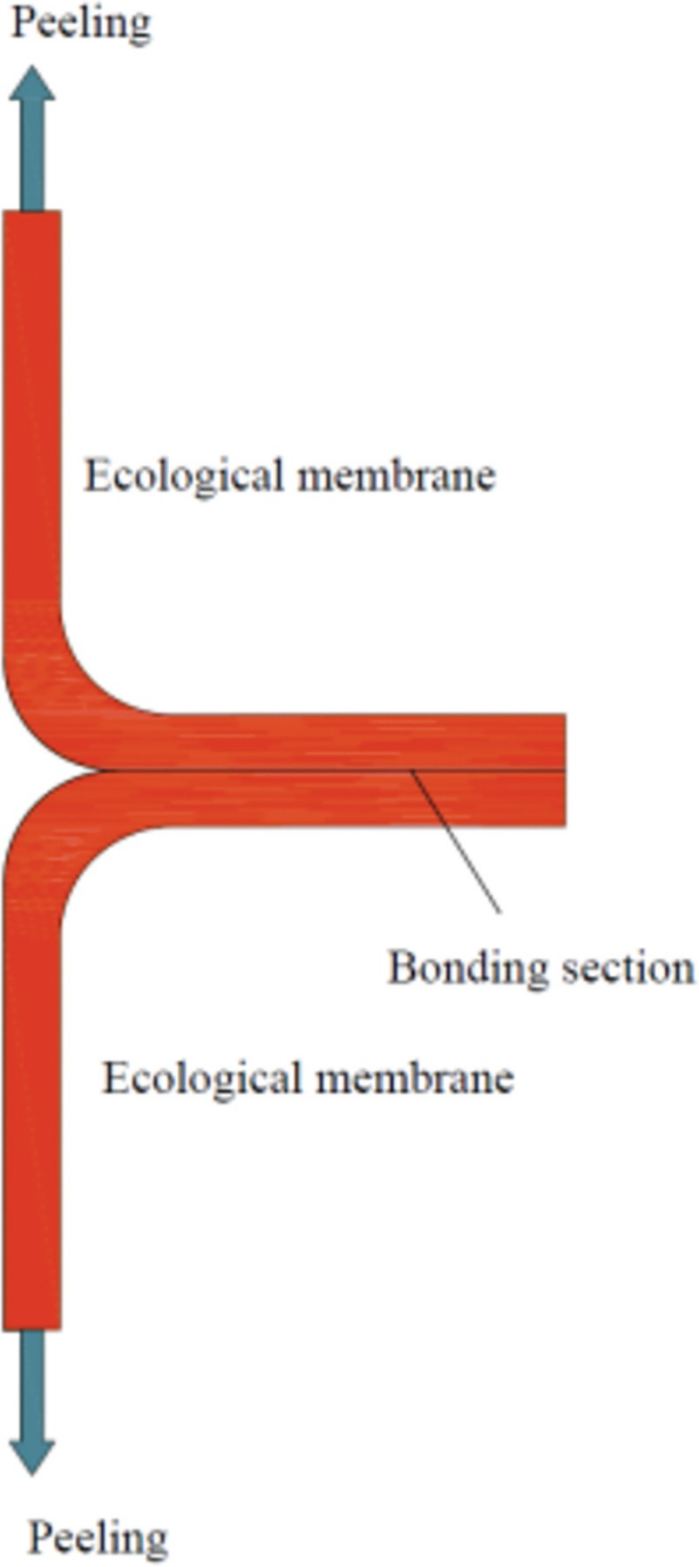
T-peel.

The viscosity of the ecological membrane surface plays an important role in soil and slope reinforcement. A viscosity test was used to test the surface viscosity of the ecological membrane samples and to study the relationship between the viscosity and material proportions of the sample. A T-peel (as shown in Fig 4) was used in this test. Two ecological membrane samples were stuck together referring to the standard test method of ASTM D5170-98 (2021) peeling strength. By applying tension at the end of the sample, the sample was peeled along the sticking line. The adhesive force on the material surface was equal to the applied peeling force. The YH-LLA-200KG universal tensile testing machine was used to apply the peeling force. The six ecological membrane samples (as shown in Table 3) were cut and developed into 120 mm long and 30 mm long strips for the viscosity tests. Three parallel tests were conducted for each sample, and the average value was taken as the final result.

The peeling strength value of the sample is calculated as follows:

$$\sigma_{T}=\frac{F_{T}}{B},$$ (6)

where, *σ*_*T*_ is the peeling strength of the sample (N/mm), *F*_*T*_ is the peeling force (N), and *B* is the width of the sample (mm).

### 2.3 Anti-erosion test

In the anti-erosion test, the slope topsoil was simulated indoors, and the ecological membrane was used to protect the slope topsoil from erosion. Rainstorm scouring was simulated to study the role of the ecological membrane in improving the anti-erosion ability of the topsoil. The test equipment included atomizing nozzles, the simulated slope topsoil, a water storage basin, and an oven. Four atomizing nozzles were connected to tap water to simulate natural rainfall, and the nozzles could be used to adjust the simulated rainfall. A laboratory metal box with a length × width × height of 30 × 20 × 4 cm was used for the filling soil and simulated slope topsoil.

**Fig 5:**
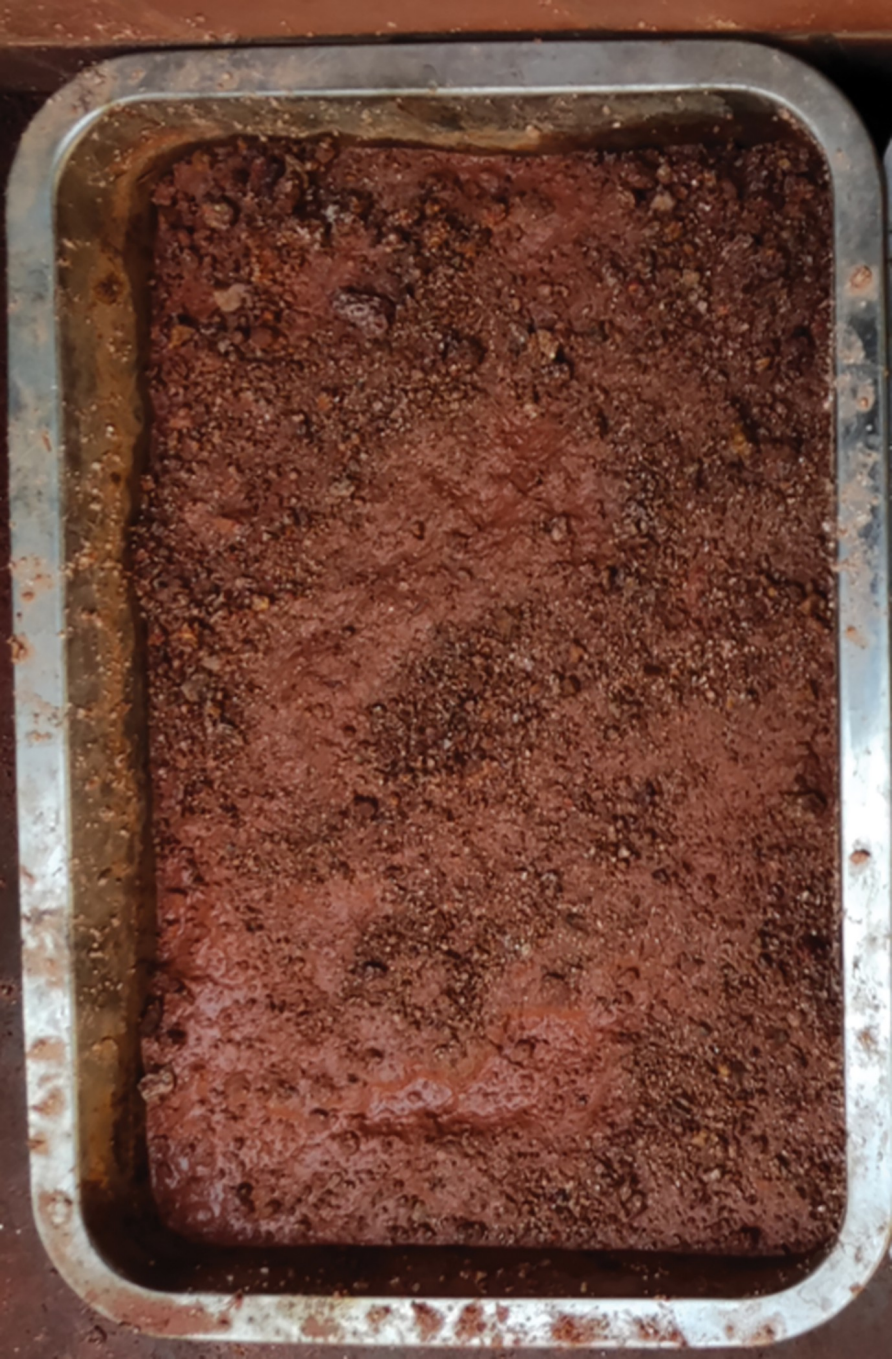
Laboratory anti-erosion test. Group 1: Soil without the protection of the ecological membrane.

**Fig 6:**
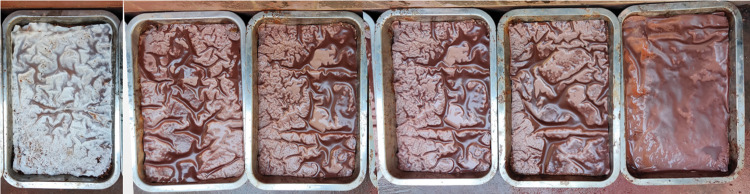
Laboratory anti-erosion test. Group 2–7: Soil without the protection of the ecological membrane.

Seven test groups were set up. In particular, 1500 g of dry sandy loam was placed in a metal box and compacted to a thickness of 3 cm. A group without the protection of the ecological membrane was set up as the control group. In the other six groups, the simulated slope topsoil was covered with the six different ecological membranes (as shown in Table 3) and compacted. Water was sprayed evenly (1.5 L/m^2^) on the surfaces of the seven test groups with a nozzle. The samples were then placed in room temperature for 72 h for testing, as shown in Figs 5 and 6, for the indoor simulated slope topsoil, and each one was designed to tilt at 70°, and a water storage basin was set below it to collect soil from erosion. The collected soil from the erosion was dried in a laboratory oven and then weighed. The simulated rainfall intensity of this test was 150 mm/h, and test time was 10 min. The erosion rate was used to measure the anti-erosion effects of the test groups. The smaller the erosion rate, the stronger was the anti-erosion ability of the slope topsoil and more effective was the topsoil protection of the ecological membrane. The calculation formula for the erosion rate is as follows:

$$EA=\frac{\Delta m}{m}\times 100\%,$$ (7)

where *Δm* is the mass of the soil from erosion after drying (g) and *m* is the weight of the dry soil in the metal box before the test (g).

### 2.4 Plant growth test

**Fig 7:**
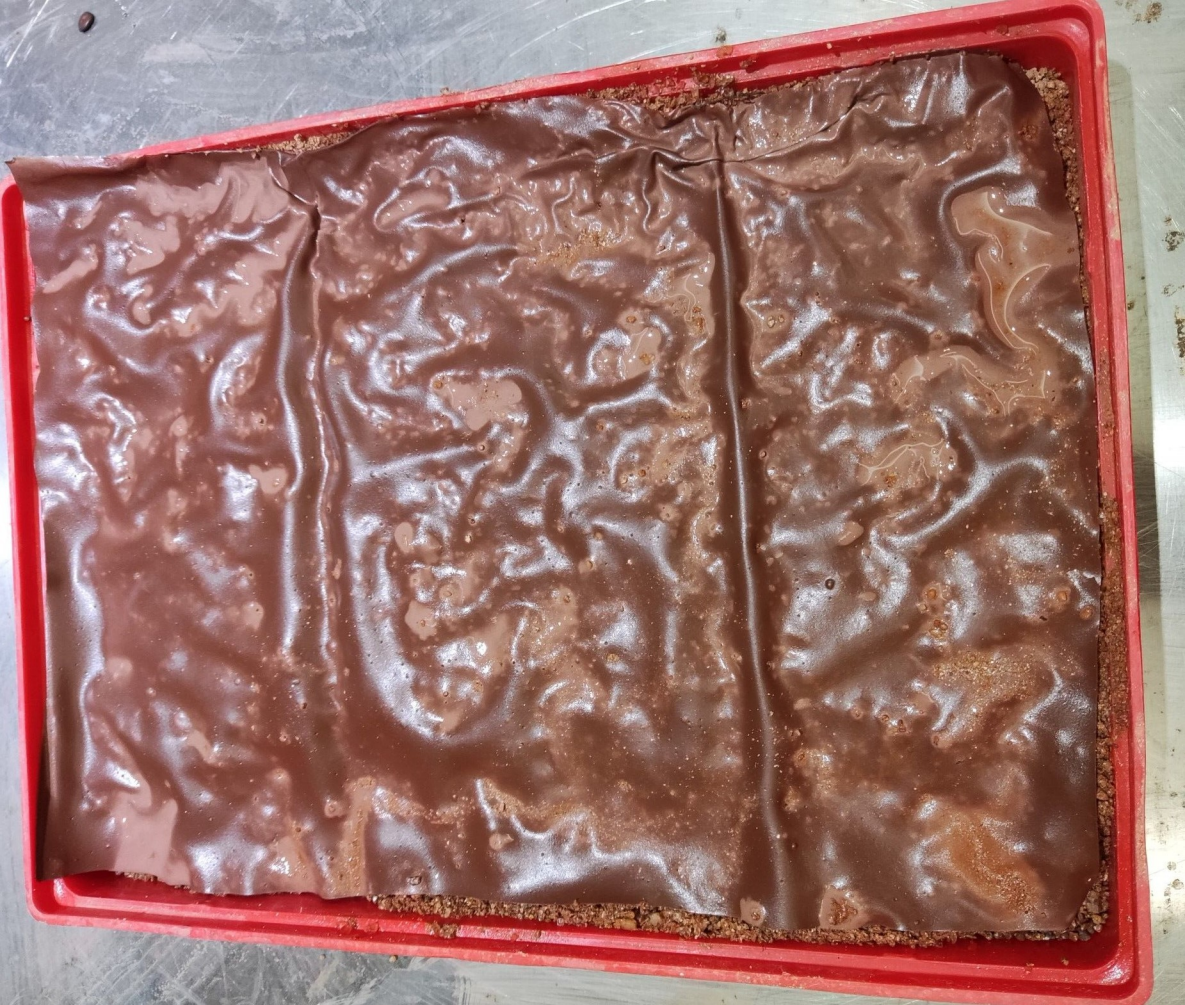
Plant growth test container.

A plant growth test [23] was conducted to observe the effects of soil mulching by the ecological membrane on the plant germination time, germination rate, and plant height and to study the promotion of the ecological restoration of the slope. Pigeonpea, a common soil restoration plant, was used in this test. Two groups of soil were designed: mulched by the ecological membrane and non-mulched. For each group, soil samples of the same quality were weighed and placed in two plastic boxes. The mulched group is shown in Fig 7; the soil was mulched by the ecological membrane. A plastic membrane seeder was used to drill holes in the ecological membrane, and the seeds were evenly seeded into the soil through the holes in the ecological membrane. In the non-mulched group, the seeds were directly seeded on the soil. The two groups were equally seeded with 30 seeds. Each group was then watered with 200 ml of water every day for the first 3 days and once every 3–5 days for the next 17 days to simulate drought. After seed germination, the germination was recorded every other day. The growth height of the plants was measured and recorded for 20 days. A parallel group was designed for each group to reduce any accidental error in the test, and the results were averaged.

The germination rate of the plants is calculated as follows:

$$\chi =\frac{a}{a_{0}}\times 100\%,$$ (8)

where *χ* is the plant germination rate of the group (%), *a* is the number of plant germinations, and *a*_*0*_ is the number of seeds sown in the group.

## 3 Results

The ecological membrane exhibits outstanding slope topsoil protection performance; it effectively improves the anti-erosion ability of the slope depending on its strength and viscosity. The ecological membrane is mulched on the slope to prevent it from being directly washed by rainwater, and simultaneously, it is adhesive to the surface soil particles through its own viscosity such that the slope can form an anti-erosion surface with strong stability and integrity. Therefore, the strength and viscosity of the ecological membrane materials are the key factors affecting the slope protection effect of the ecological membrane.

### 3.1 Tensile strength of ecological membrane

**Fig 8:**
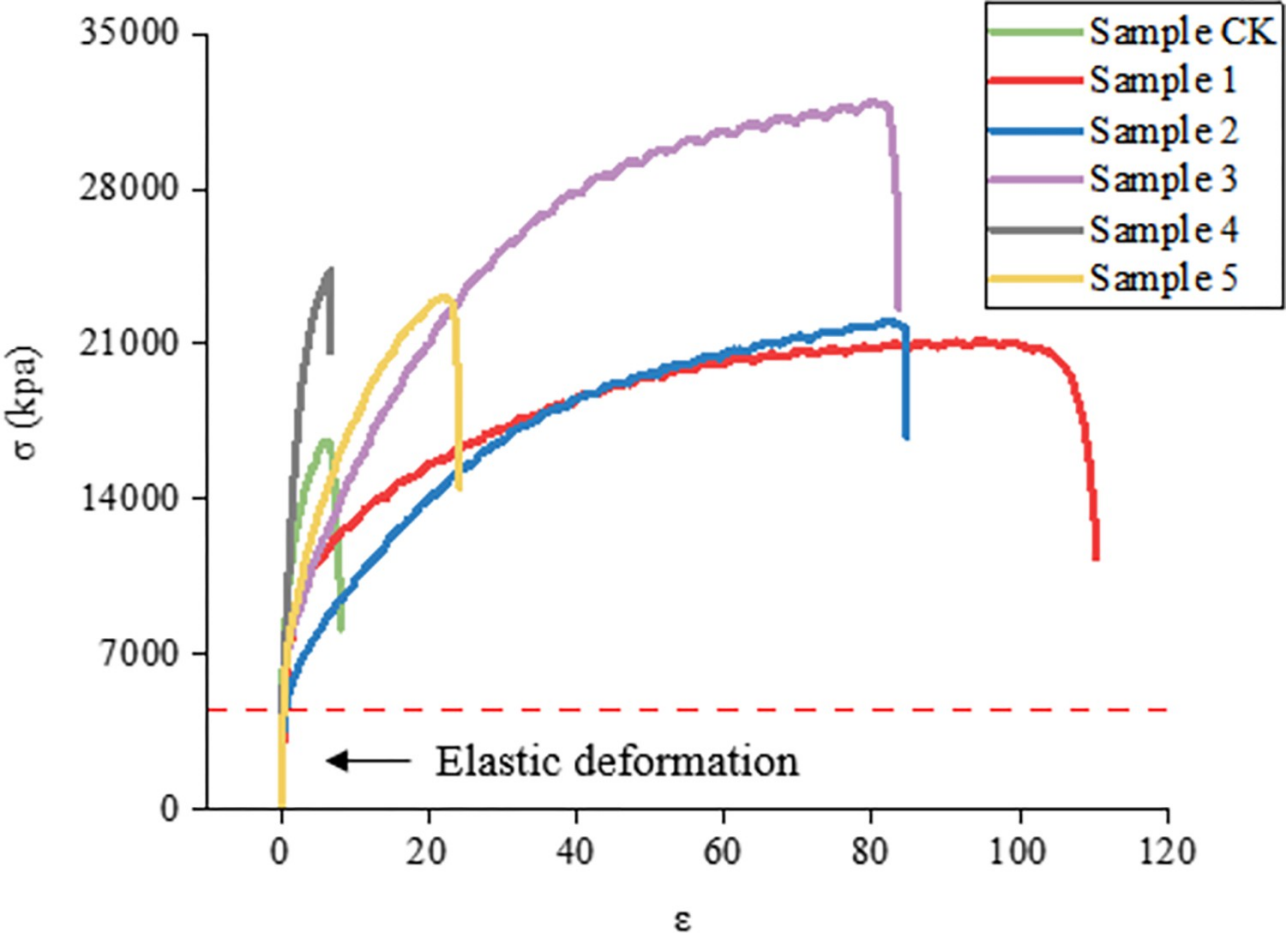
Stress–strain curve of ecological membrane sample.

The stress–strain curve of an ecological membrane sample can reflect its deformation and failure characteristics under an external force, and the mechanical properties of the sample can be understood through the curve. The stress–strain curves of several ecological membranes are depicted in Fig 8. It can be seen that the ecological membrane samples have similar deformation and damage characteristics, displaying evident elasticity and plasticity. The deformation length before damage is large. Generally, the samples are soft and tough. According to Fig 8, the stress in the first stage of the tensile deformation process of the several samples is proportional to the stress of the sample; this satisfies Hooke’s law and presents a linear relationship. The sample is in elastic deformation, hence, the Young’s modulus of elasticity *E* of the sample can be calculated (indicating the rigidity of the material).

**Fig 9:**
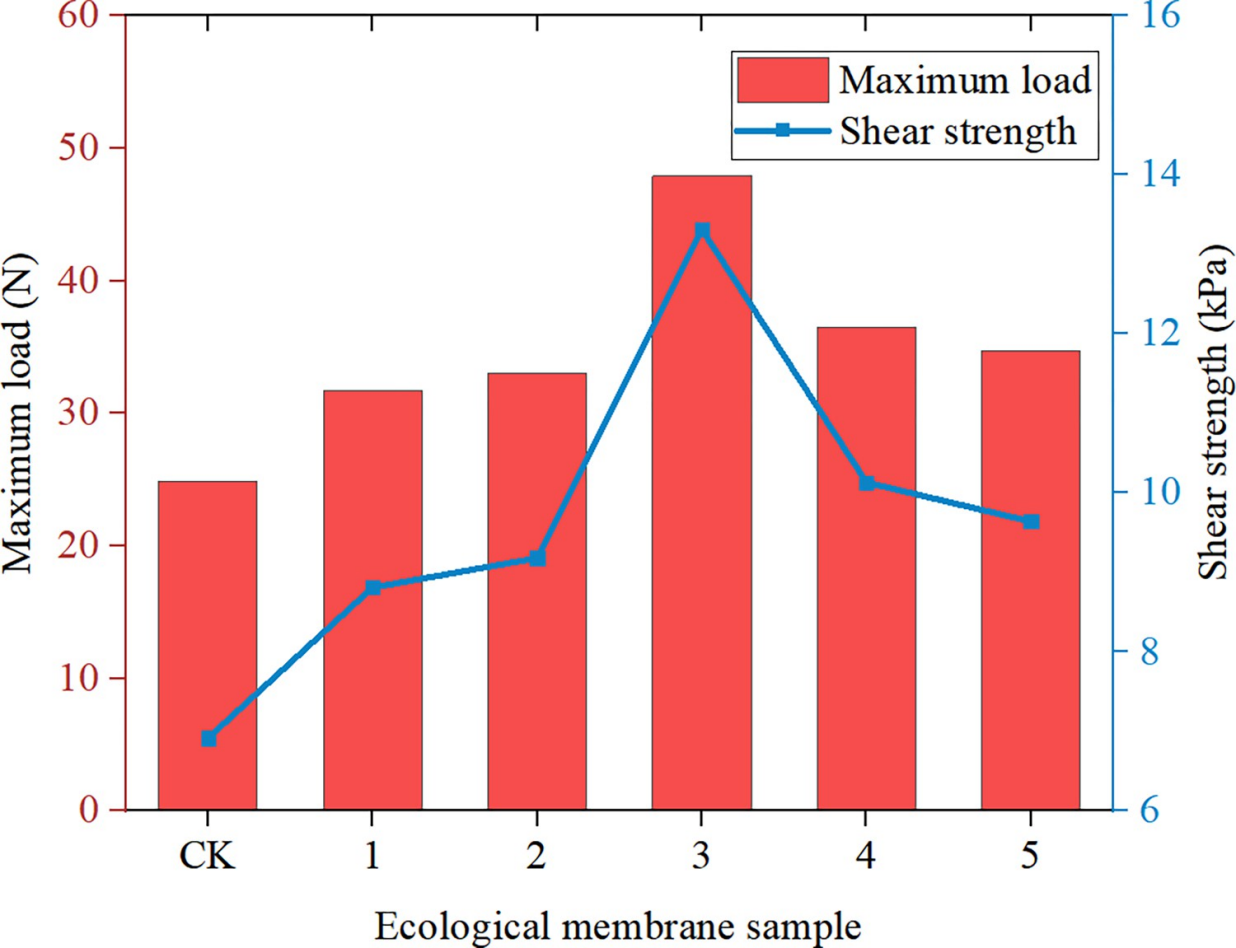
Maximum load and shear strength of the ecological membrane samples.

**Fig 10:**
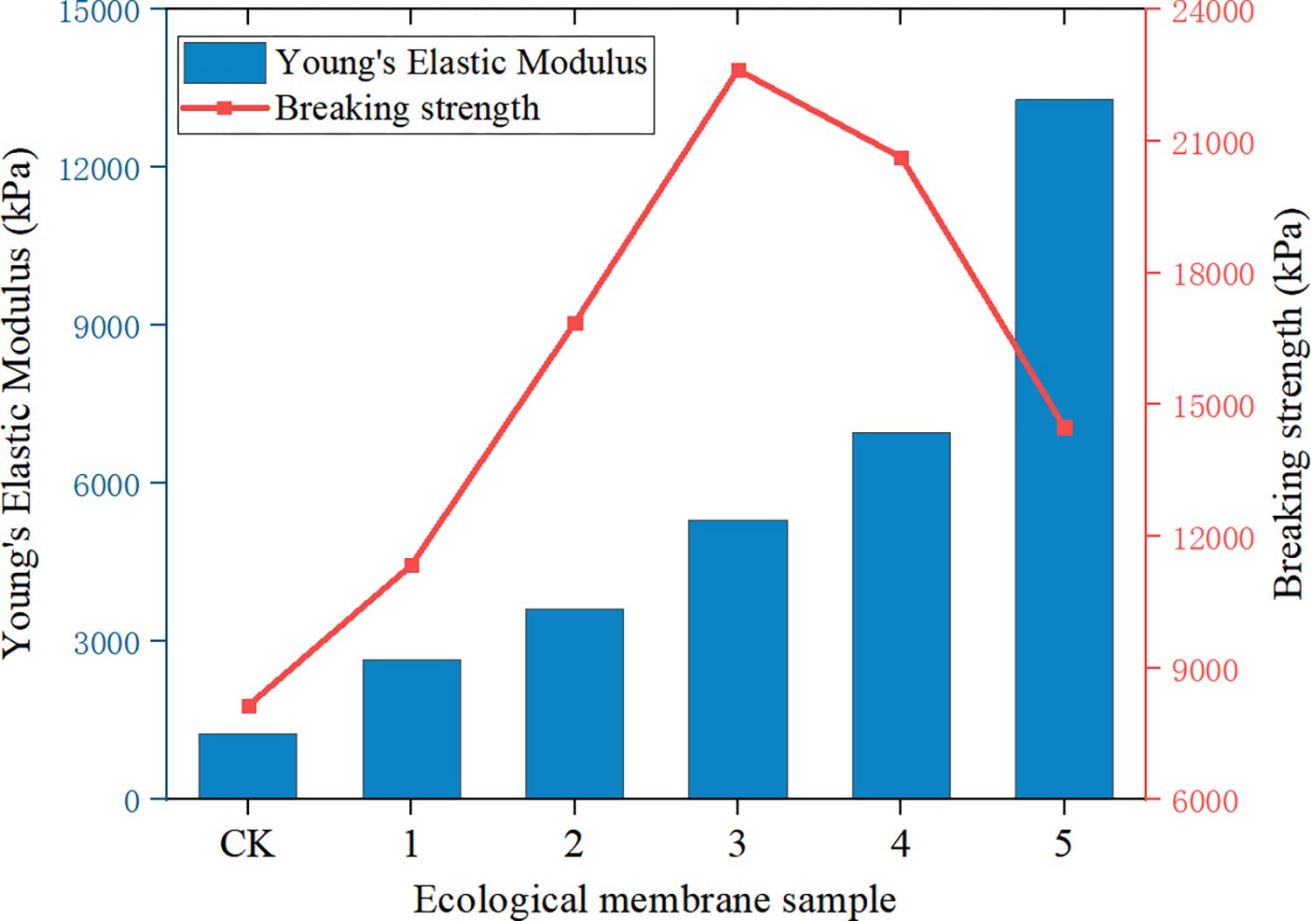
Young’s modulus of elasticity and breaking strength of the ecological membrane samples.

**Fig 11:**
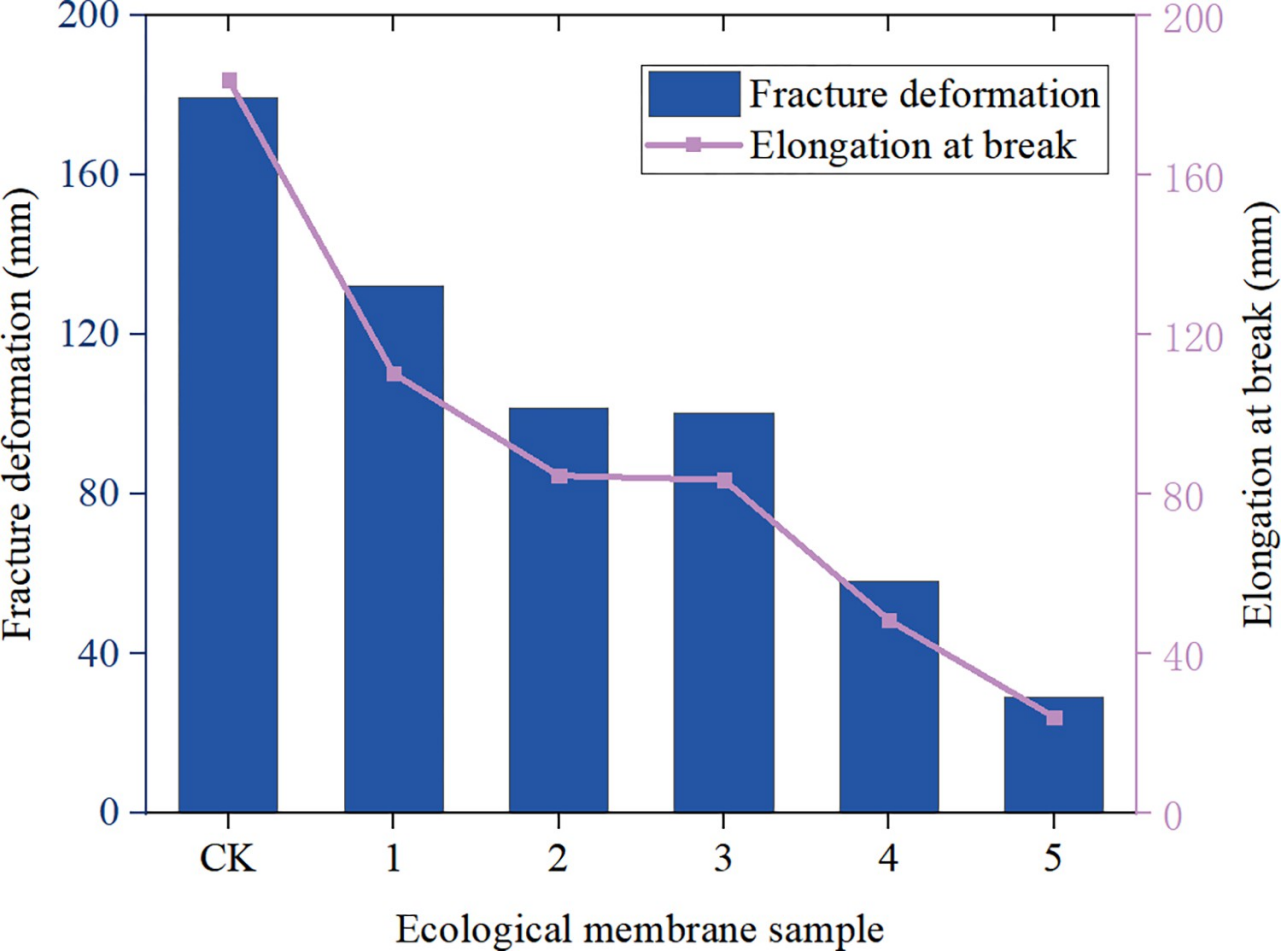
Fracture deformation and elongation of the ecological membrane samples’.

Figs 9–11 depicts the maximum load, breaking strength, breaking deformation, elongation *ε*, shear strength, and Young’s modulus of elasticity of the six ecological membrane samples. The larger the Young’s modulus *E*, the harder the sample. The larger the value of *σ*_*b*_, the stronger the sample. The larger the value of *ε*_*b*_, the tougher the sample. By combining Figs 8–11, the tensile strength test results of the ecological membrane can be obtained.

The similarities of the six ecological membrane samples with different material ratios (as shown in Table 3) are as follows.

1. It can be seen from the stress–strain curves of the ecological membrane samples in Fig 8 that there is no evident yield point during the tensile deformation of the sample, and the deformation and break of the sample mainly show elastic–plastic deformation until the fracture and breaking of the sample. The material shows elastic–plastic characteristics.
2. It can be seen from Figs 9 and 10, which depict the mechanical properties of the ecological membrane samples, that the maximum load and breaking strength of the samples are large (the strength of the six samples are high).
3. It can be seen from the mechanical properties of the ecological membrane shown in Fig 11 that the deformation capacity of the ecological membrane is strong.

In summary, the ecological membrane demonstrates toughness and tenacity.

The differences between the six ecological membrane samples with different material ratios are as follows.

1. Upon comparing the maximum tensile loads, shear strengths, and fracture strengths of the samples in Figs 9 and 11, it can be seen that the strengths of the five samples with red bed soil exceed that of the control group sample without red bed soil. The strength of sample 3 (with 30% red bed soil) is the largest, and the strength of sample CK (without red bed soil) is the weakest.
2. In Fig 11, upon comparing the elongation at the breaking of the samples, it can be seen that with an increase in the amount of red bed soil, the deformation capacity of the samples become weaker and the sample becomes more brittle. Sample CK (without red bed soil) is the most tenacious, and its deformation capacity is the strongest, and the sample 5 (with 50% red bed soil) is the most brittle.
3. In Fig 10, upon comparing the Young’s modulus of elasticity of the samples, it can be seen that with an increase in the amount of red bed soil, the sample becomes tougher. The sample CK without red bed soil is the softest, and the sample 5 with 50% red bed soil is the toughest.

### 3.2 Viscosity of ecological membrane

**Fig 12:**
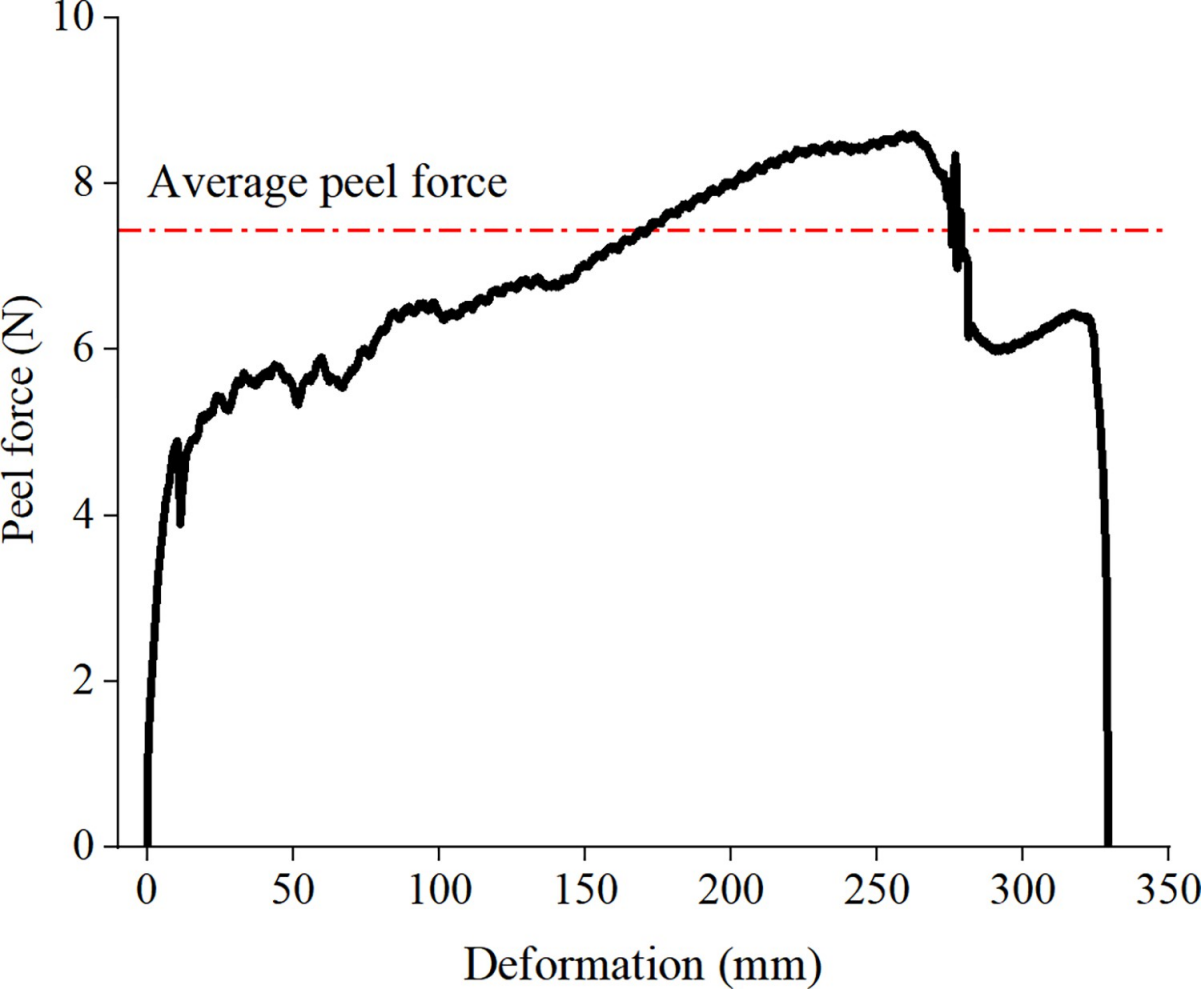
Deformation–peeling strength curve of the ecological membrane peeling test. Ecological membrane sample CK.

**Fig 13:**
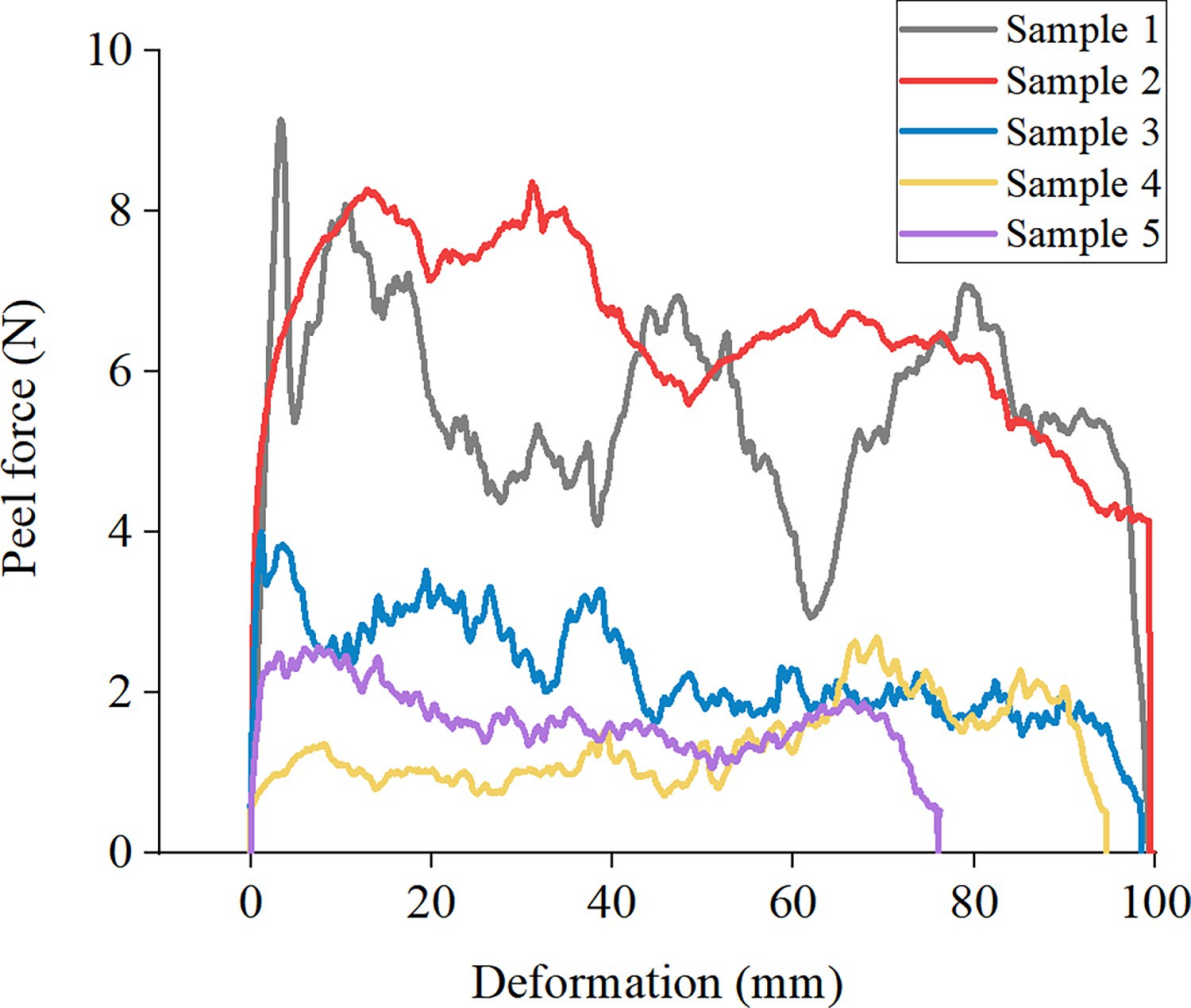
Deformation–peeling strength curve of the ecological membrane peeling test. Ecological membrane samples 1–5.

The results from the peel test are shown in Figs 12 and 13 and Table 5. It can be seen from Fig 12 that the deformation–peeling strength curve of the ecological membrane without red bed soil is more well-distributed than those of the ecological membranes with red bed soil, with less curve fluctuations and a stable change in surface viscosity. So the average viscosity of the ecological membrane CK is greater than that of the other samples.

**Table 5:** Results of peeling test.

Ecological membrane sample	Content red bed soil	Maximum peeling force (N)	Average peeling force (N)	Peel strength (N/mm)
CK	0%	8.601	7.436	0.744
1	10%	7.08	5.37	0.537
2	20%	6.757	4.932	0.493
3	30%	3.324	2.224	0.222
4	40%	2.688	1.424	0.142
5	50%	1.992	1.554	0.155

It can be seen from Table 5 that with an increase in the amount of red bed soil in the ecological membrane, the peel strength of the ecological membrane, that is, the viscosity, generally decreases. Among the samples, the ecological membrane without red bed soil has the strongest viscosity, with a maximum viscosity of 8.61 N. The ecological membrane with 50% red bed soil has the lowest maximum viscosity, with a maximum viscosity of 1.992 N. The average peel strength of the ecological membrane, that is, the unit viscosity of the ecological membrane, also shows a trend of weakening with an increase in the amount of red bed soil.

It can be seen from the above analysis of the test results that the more composite polymer adhesive materials contained in the ecological membrane, the stronger is its viscosity and the more red bed soils contained in the ecological membrane, the weaker is its viscosity. It shows that the main factor contributing to the viscosity of the ecological membrane is the composite polymer adhesive material, not the red bed soil. The sticky ecological membrane can bond with the soil particles on the surface. The viscosity of the ecological membrane also plays an important role in protecting the slope soil from erosion. It is an important factor in determining the application performance of the ecological membrane with respect to slope protection, as it can quickly form a stable and integral erosion-resistant surface for the soil, effectively reducing the erosion rate of the slope.

### 3.3 Anti-erosion performance of ecological membrane

**Fig 14:**
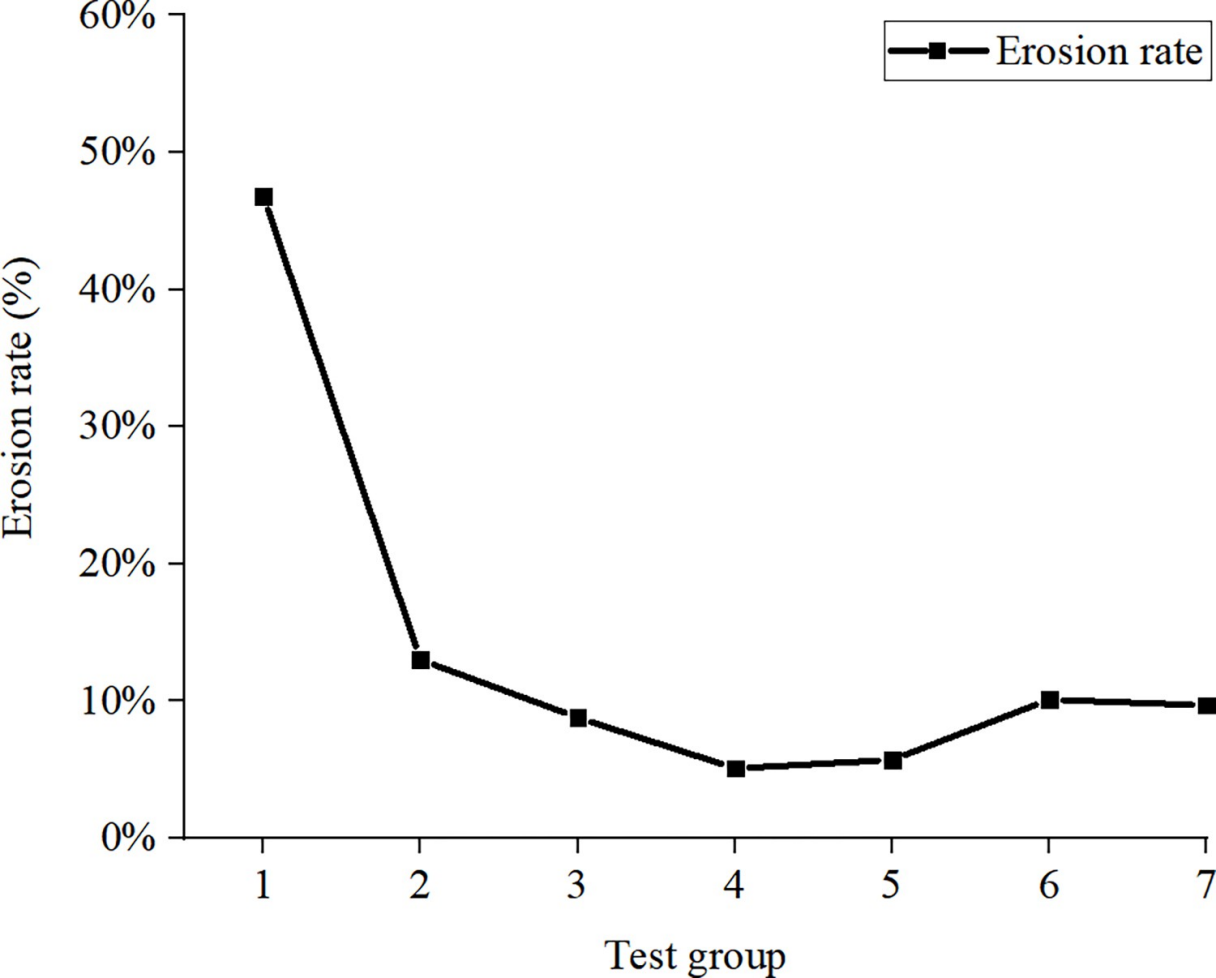
Erosion rate.

It can be seen from the erosion rate of the slope in Fig 14 that after the slope is mulched with the ecological membrane, the erosion rate of the slope decreases and the anti-erosion ability of the slope is improved; the ecological membrane displays good soil consolidation and slope protection performance. For the control group (group 1) without the protection of ecological membrane, the 10-min slope erosion rate is 46.8%. After using the ecological membrane for slope protection, the erosion rate of the slope is significantly reduced. The 10-min erosion rate of the slope with the protection of the ecological membrane without red bed soil (group 2) is 13%, which is less than 1/3 of that of the control group. Moreover, when using the ecological membrane with red bed soil in slope protection (group 3–7), the erosion rate of the slope is less than that of the group using the ecological membrane without red bed soil. The ecological membrane with red bed soil shows a better improvement in the anti-erosion effect; the erosion rates of groups 3 and 4 (under the protection of ecological membrane with 20% and 30% red bed soil, respectively) are the lowest, exhibiting the best slope protection effect. It can be seen from Fig 7 that the mulching of the slope with the ecological membrane can alleviate the direct scouring of the soil by rainwater and enhance the anti-erosion ability of the slope. The surface of the ecological membrane is sticky, and the wet ecological membrane is adhesive to the soil particles on the slope surface, thereby enhancing the compactness of the soil layer on the slope surface. Under the effect of the viscosity of the ecological membrane, when the slope is washed by rain, the number of soil particles taken away by the water flow can be greatly reduced.

### 3.4 Results of plant growth test

Water and temperature are crucial factors for plant germination and growth. The ecological membrane mulching plays a significant role in the water and moisture conservation of the soil, thus affecting the growth of the soil plants.

**Fig 15:**
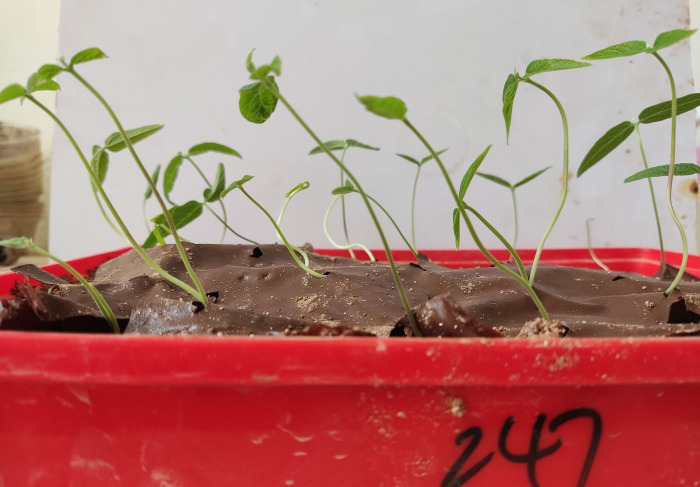
Plant growth condition.

As shown in Table 6 and Fig 15, the results from the plant growth test demonstrate that compared with the non-mulched test group, the germination rate of the group mulched with the ecological membrane significantly improves from 73% to 90%, the plant height improves from 76.7 cm to 22.8 cm, and the germination time of the plant seeds is shortened from 5 days to 3 days. In summary, the ecological membrane mulching promotes the growth and development of plants in the soil, with good ecological effects.

**Table 6:** Result of plant growth test.

Group Parameter	Mulched with ecological membrane	Non-mulched
Germination	90%	73%
Germination time	3 days	5 days
Plant height	22.8 cm	16.7 cm

## 4 Discussion

### 4.1 Effect of the materials addition of ecological membrane on its properties

From the tensile strength test of the ecological membrane, it can be seen that adding red bed soil into the ecological membrane can improve its strength and that the addition of 30% red bed soil is the optimal ratio for improving the strength of the ecological membrane samples. The strength increase of the samples slows down when the addition of the red bed soil exceeds 30%. The addition of the mineral components of the red bed soil can improve the strength of the ecological membrane; they are responsible for reinforcing the ecological membrane, which can be equated to the role of the skeleton of the ecological membrane. However, the ecological membrane mainly relies on the viscosity of composite polymer adhesive materials to form a tight and firm structure. When an excessive amount of red bed soil is added and too few composite polymer adhesive materials are used, the adhesive force provided by the composite polymer adhesive materials is insufficient, and the strength of the ecological membrane is correspondingly weakened. The viscosity of the composite polymer adhesive also is the source of the elastoplasticity and deformation capacity of the ecological membrane. Thus, the addition of the mineral components of the red bed soil makes the ecological membrane brittle and tough. The greater the addition of red bed soil, the more brittle and tough is the membrane material, the more composite polymer adhesive materials added to the ecological membrane, the stronger is its viscosity. The main factor contributing to the viscosity of the ecological membrane is the composite polymer adhesive material, rather than the red bed soil.

### 4.2 Influence of mechanical properties of ecological membrane on its slope protection performance

**Fig 16:**
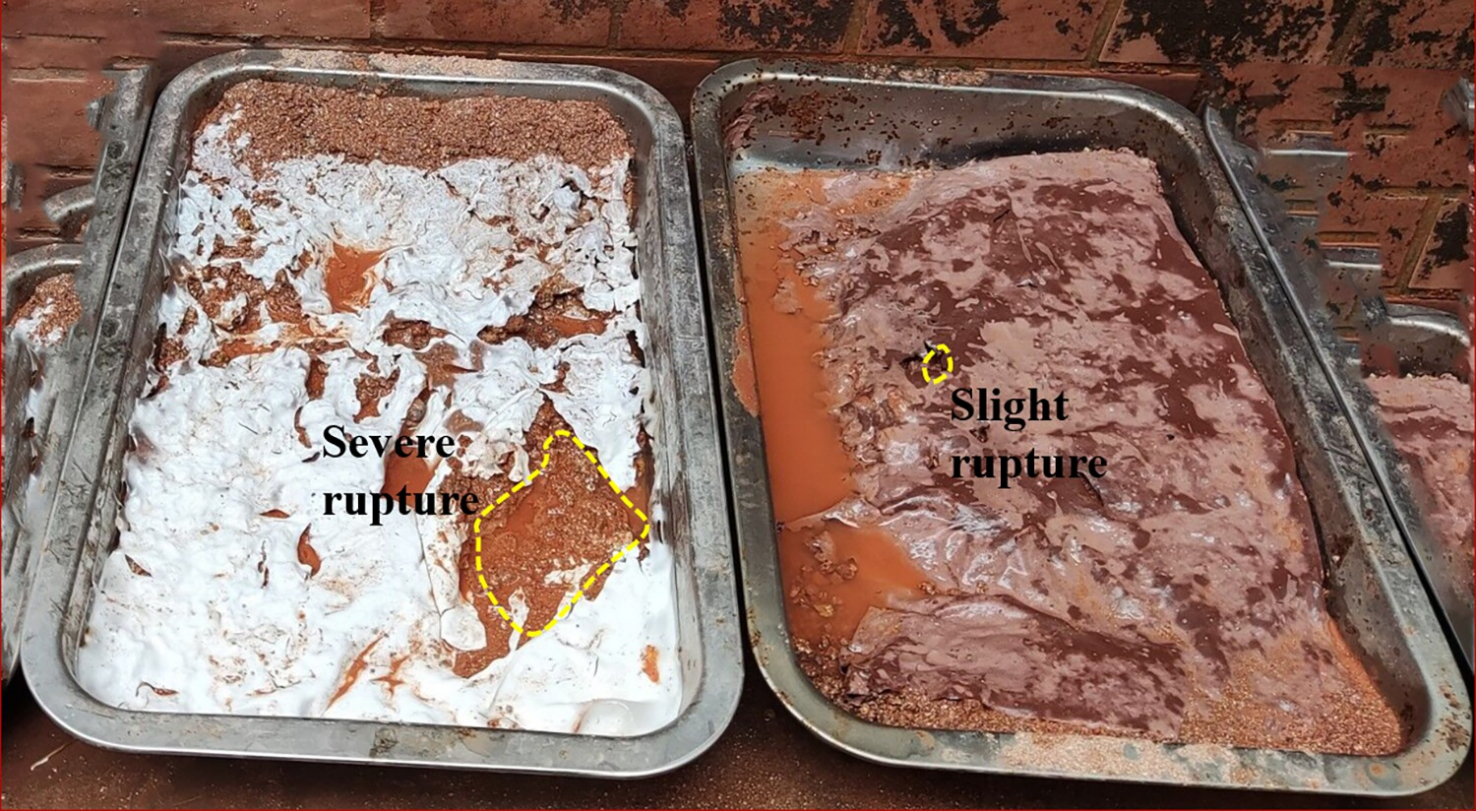
Soil after 10 min scouring under the protection of the ecological membrane. Under the protection of the ecological membrane samples CK and 1.

**Fig 17:**
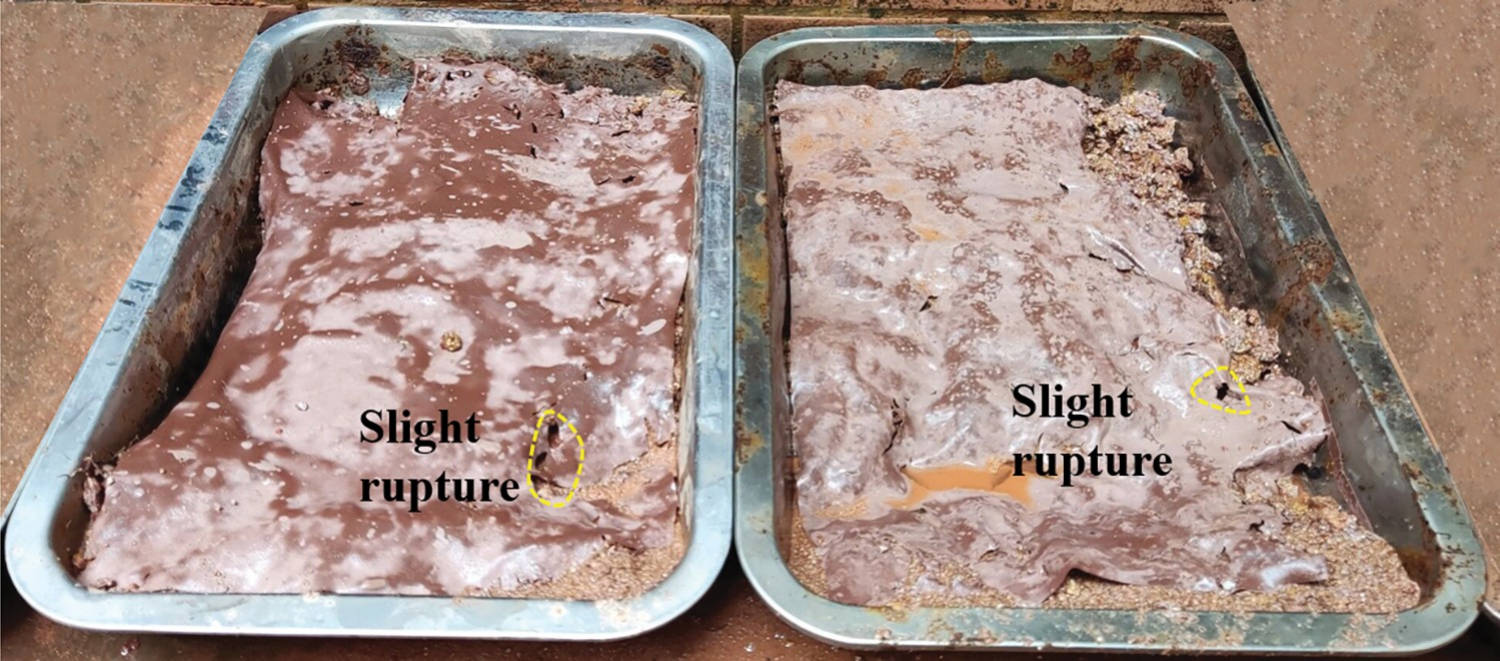
Soil after 10 min scouring under the protection of the ecological membrane. Under the protection of the ecological membrane samples 2 and 3.

**Fig 18:**
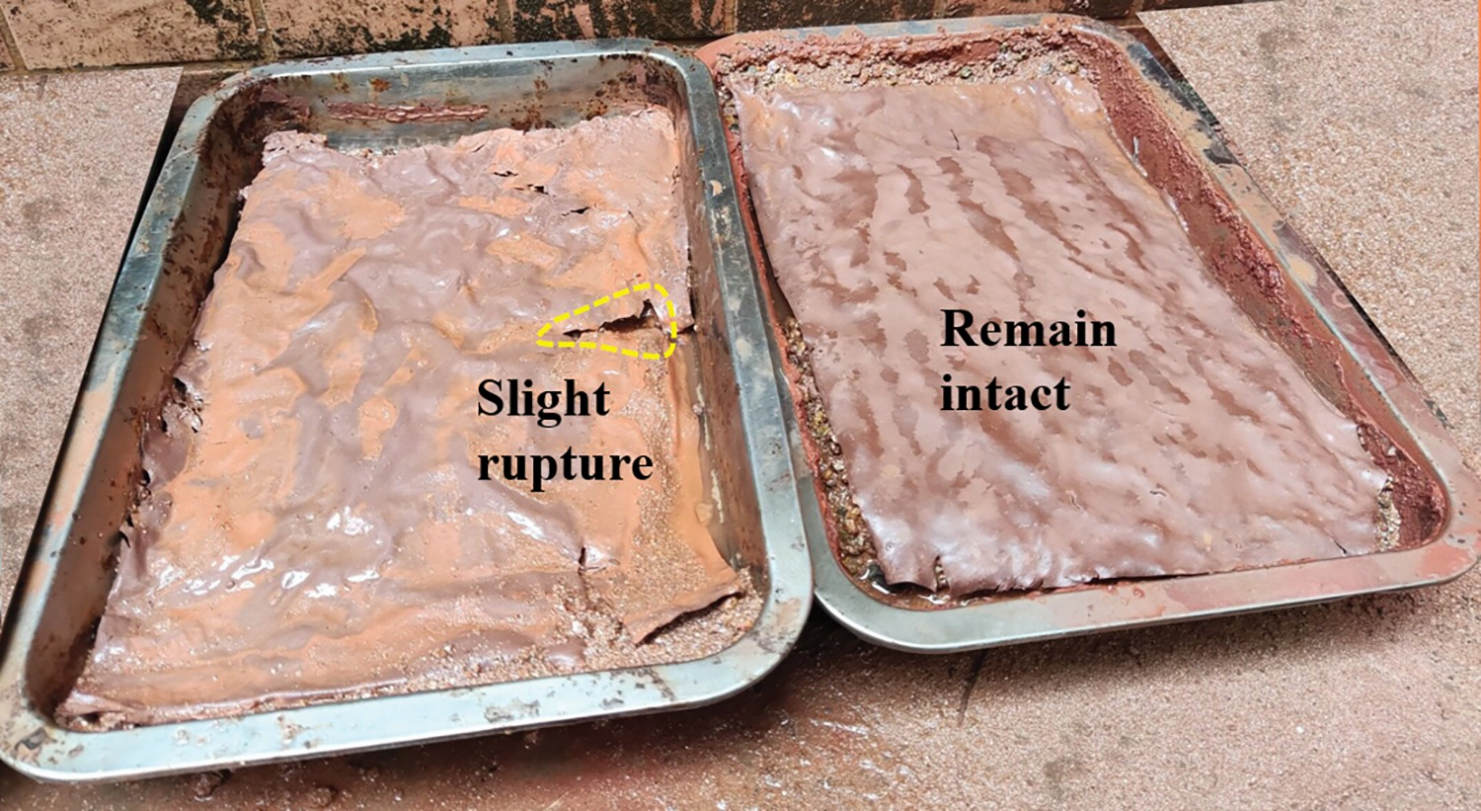
Soil after 10 min scouring under the protection of the ecological membrane. Under the protection of the ecological membrane samples 4 and 5.

The strength and viscosity of the ecological membrane play an important role in supporting its slope protection performance. Figs 16–18 shows the state of the slope after 10 min of scouring in the anti-erosion test groups 2–7. Through comparison, it is found that the weak ecological membrane without red bed soil in test group 2 breaks down after rainstorm scouring, leading to its weak protection effect on the slope. The ecological membrane with red bed soil is stronger, and no evident fracture occurs after 10 min of rain scouring; thus, its protection effect on the slope is stronger. It can be seen that the breaking strength of the ecological membrane sample CK is insufficient, in order to protect the soil from heavy rain erosion, the breaking strength of the ecological membrane should be greater than or equal to the breaking strength of ecological membrane sample 1, that is, 8140 kPa.

**Fig 19:**
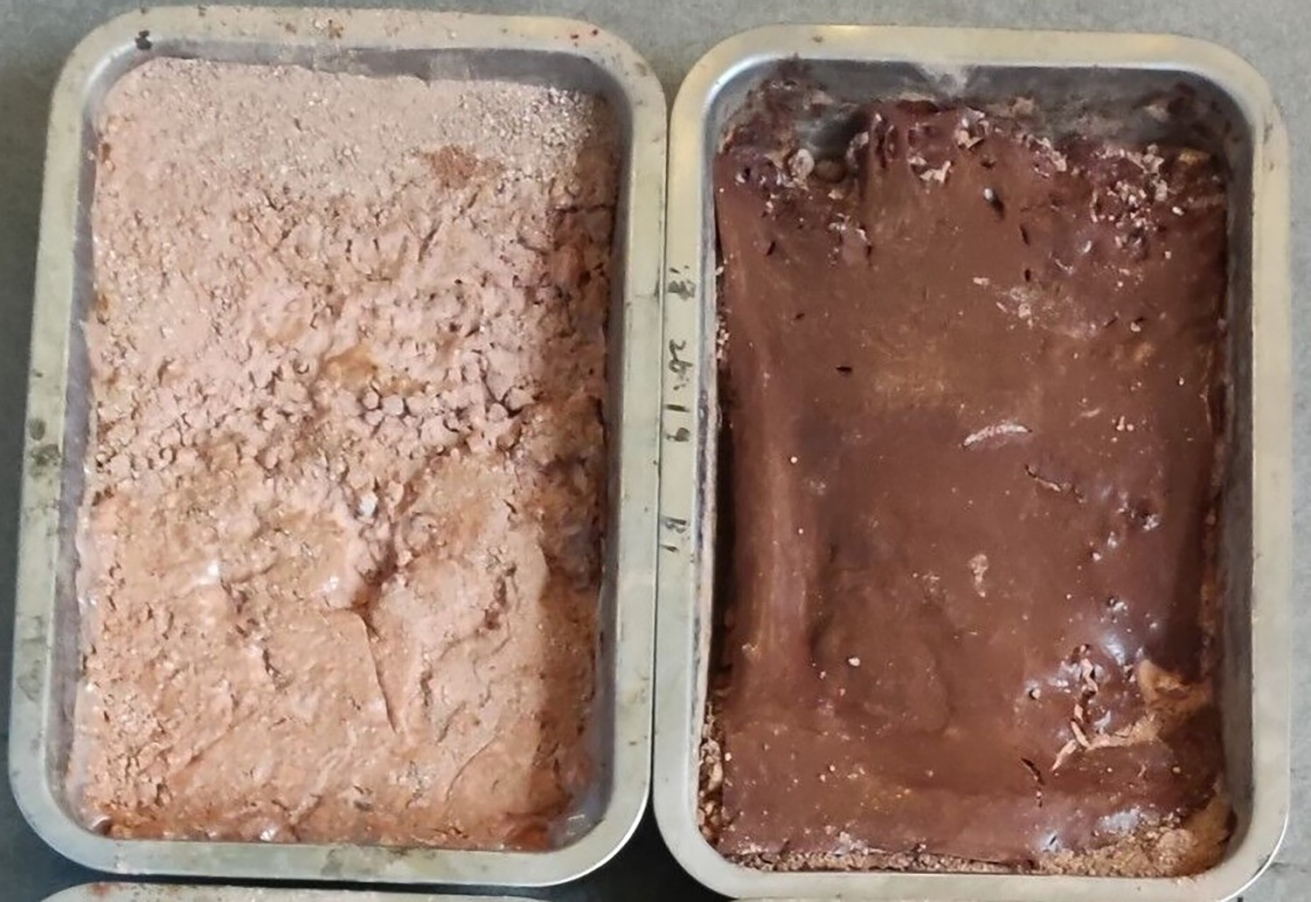
Anti-erosion test group after drying. Under the protection of the ecological membrane samples CK and 1.

The ecological membranes samples covered on the slope were softened after being washed by the rain and were adhesive with the soil through their viscosity. After the rain, the ecological membranes were dried, and the mechanical properties were restored after drying. As shown in Fig 19, the samples are restored to membranes with strong tensile strength. The membranes bond with the surface soil and are integrated into the soil surface such that the soil surface can form a stable and complete structure. Accordingly, the slope continues to be protected during subsequent rainfall.

**Fig 20:**
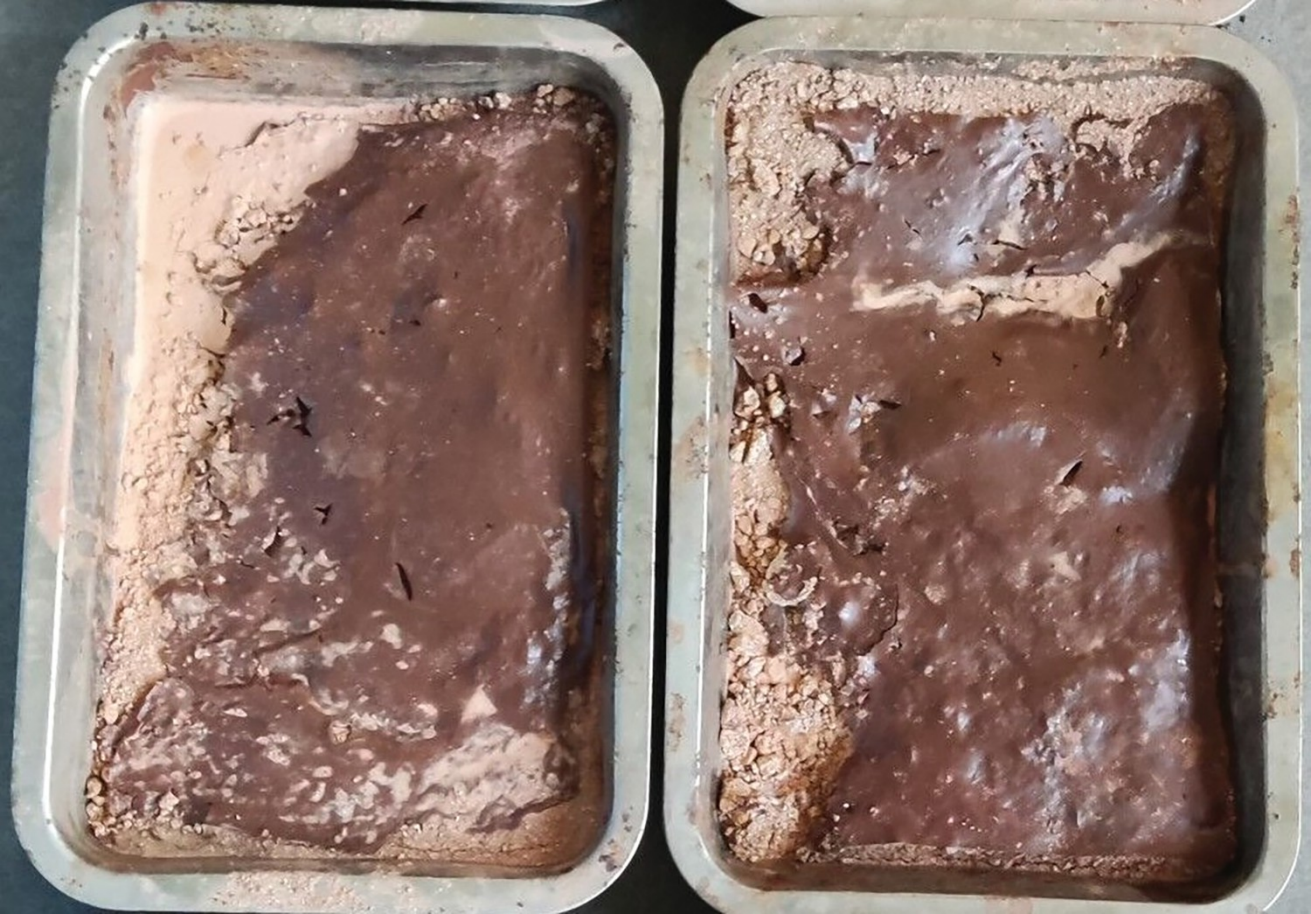
Anti-erosion test group after drying. Under the protection of the ecological membrane samples 2 and 3.

**Fig 21:**
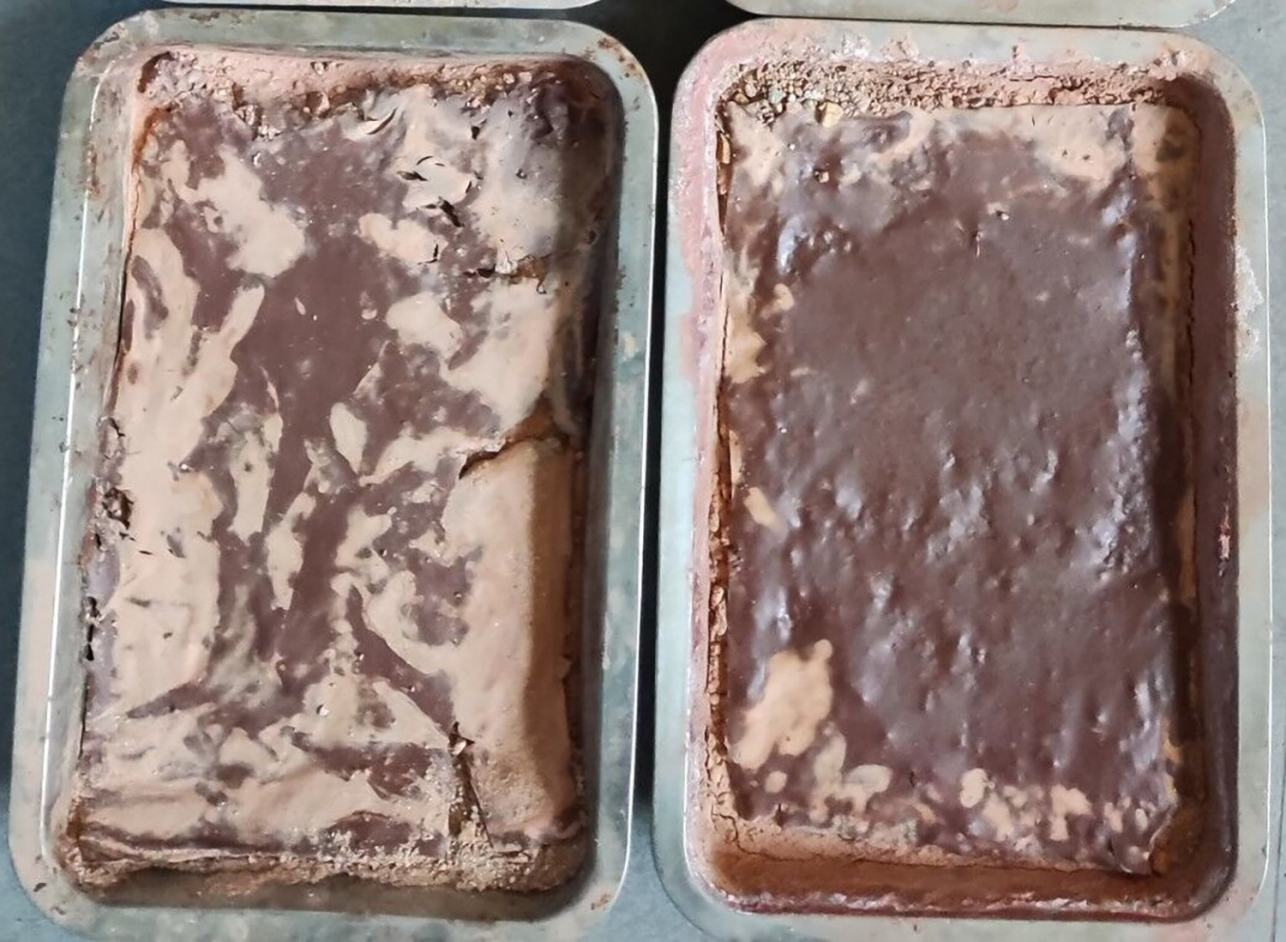
Anti-erosion test group after drying. Under the protection of the ecological membrane samples 4 and 5.

The ecological membrane can enhance the anti-erosion ability of the slope by mulching the slope soil and can consolidate and protect the soil with its strength and viscosity. Figs 19–21, from top to bottom, shows the anti-erosion test results for groups 2–7, in which test group 2 uses an ecological membrane without red bed soil for protection. This ecological membrane has the strongest surface viscosity, but its strength is weak. Therefore, it breaks after the simulated rainstorm scouring. Because its texture is the softest, it completely bonds with the surface soil after drying. Test groups 3–5 use ecological membranes with 10%, 20%, and 30% red bed soil for protection, respectively. These ecological membranes have adequate viscosity and soft textures. It can be seen that they are also well-bonded with the soil on the simulated slope surface after drying, thereby enhancing the stability of the soil on the slope surface. Test groups 6 and 7 use ecological membranes with 40% and 50% red bed soil for protection, respectively. These ecological membranes are low in viscosity and tough in texture. It can be seen that after drying, they have poor adhesion with the soil on the slope surface, and during rainstorm scouring, rainwater washes down the gap between the slope surface and ecological membrane, causing the loss of soil particles on the slope. Therefore, their protection effect is not as good as that of the ecological membranes in test groups 3–5. It can be seen that in order to make the ecological membranes better bond with soil, protect the soil from heavy rain erosion, the viscosity of the ecological membranes should be greater than or equal to the viscosity of the ecological membrane sample 3, which is 2.224 N.

**Fig 22:**
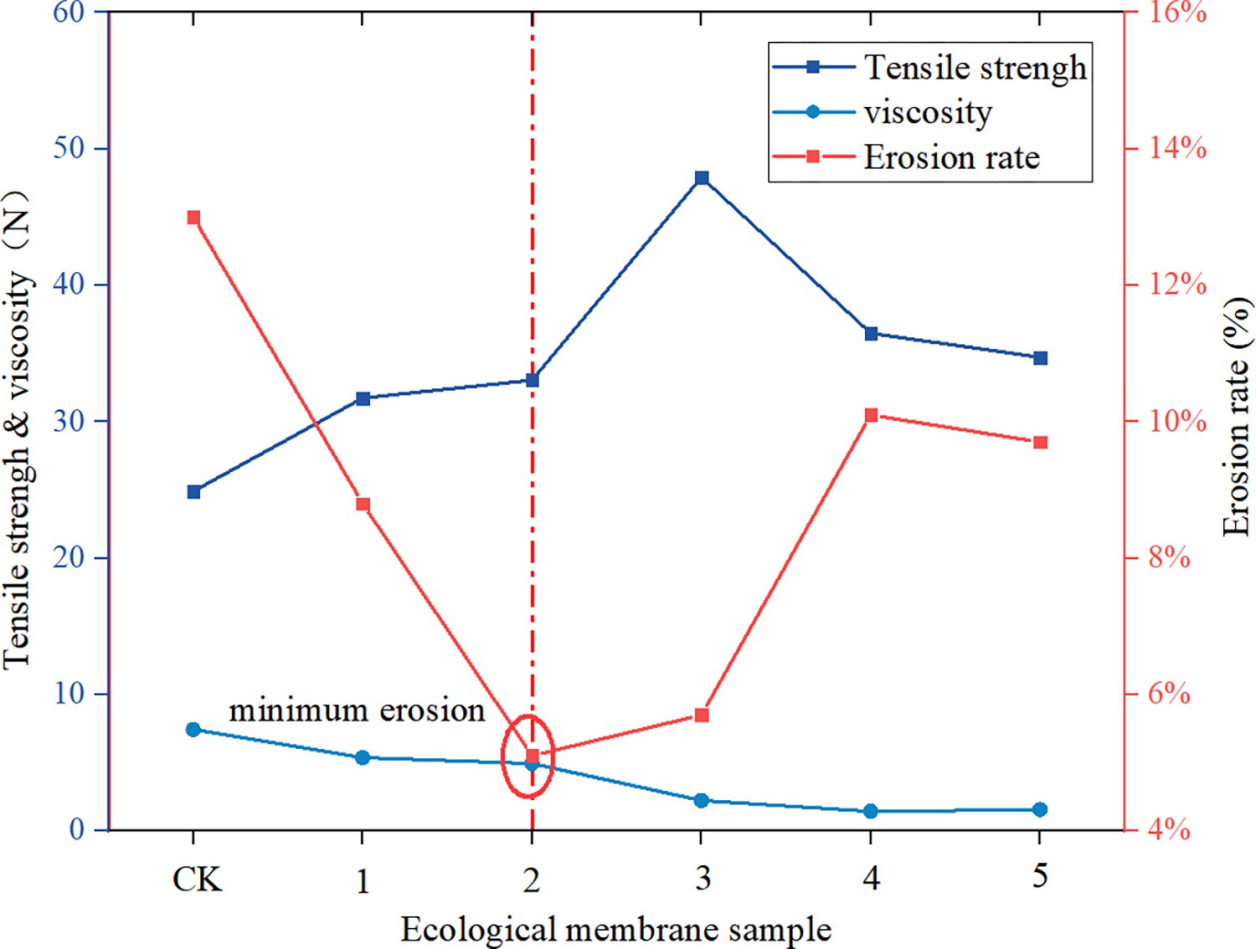
Relationship between mechanical properties and soil protection of the ecological membrane base on the test results.

The relationship between the mechanical properties and slope protection of the ecological membrane is shown in Fig 22. It can be seen that under heavy rain scouring, the strength of the ecological membrane has a greater impact on the improvement of the slope anti-erosion than its viscosity. With an increase in the strength of the ecological membrane samples, the anti-erosion of the slope covered by the samples tends to increase (the erosion rate decreases), that is, the slope protection performance of the ecological membrane samples is enhanced. When the strength of the ecological membrane starts to decrease, the anti-erosion of the slope covered by the ecological membrane decreases (the erosion rate increases), that is, the slope protection performance of the ecological membrane deteriorates. The overall change trend of the slope protection performance of the ecological membrane is most affected by its strength. However, the ecological membrane with the strongest slope protection performance is not sample 3 with the largest strength of the 30% red bed soil addition but sample 2 with medium strength. Moreover, the slope protection performances of samples 3 and 4 with stronger strength are also weaker than that of sample 2 with weaker strength. This is because the viscosity of sample 2 is stronger than that of samples 3, 4, and 5, indicating that the viscosity of the ecological membrane also significantly impacts the slope protection performance. It can be seen from the above analysis that the strength and viscosity of the ecological membrane both significantly influence the slope protection performance under the condition of rainstorm scouring.

### 4.3 Slope protection mechanism of ecological membrane

**Fig 23:**
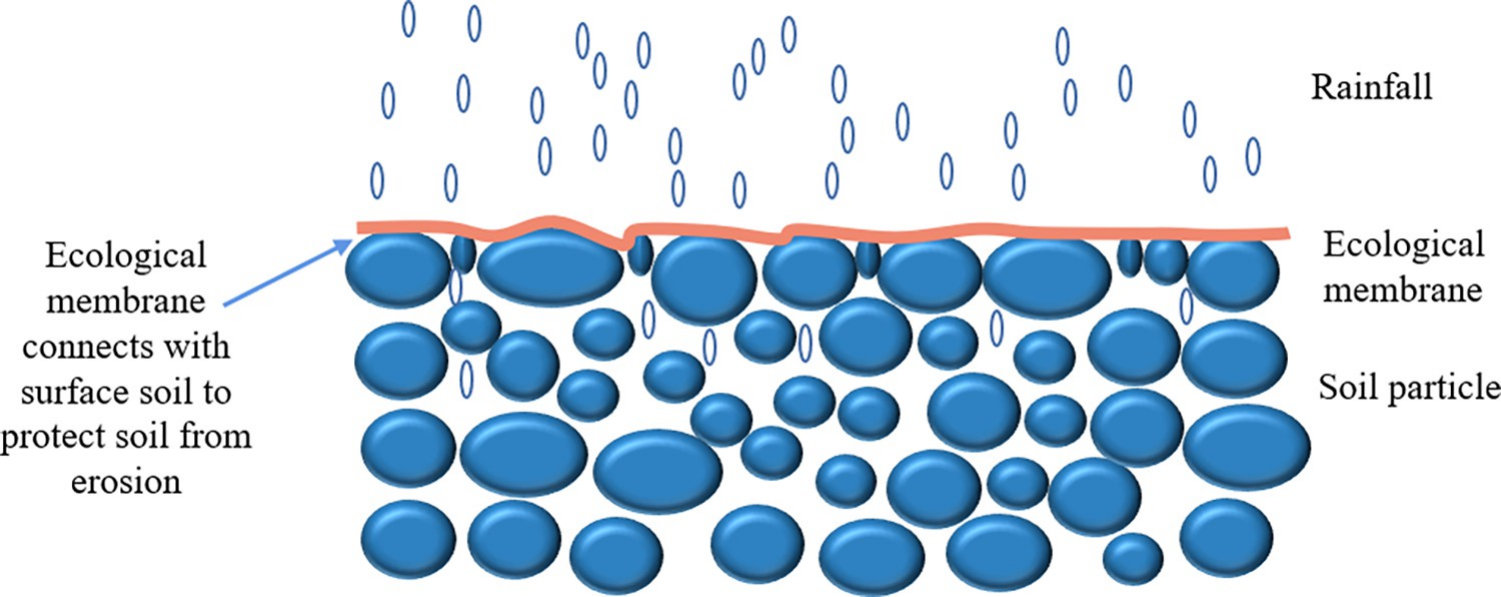
Diagrammatic sketch of how ecological membrane protects slope topsoil.

From the above research and analysis, it can be seen that the ecological membrane exhibits good slope protection and soil consolidation performances. It effectively improves the anti-erosion ability of the slope soil by relying on its own material strength and viscosity. As shown in Fig 23, the ecological membrane can protect soil particles from direct scouring by rainfall and can further reduce the occurrence of slope water and soil losses by bonding its own viscosity with topsoil particles. Moreover, the mulching of soil by the ecological membrane can effectively promote the growth and development of plants, and the ecological membrane mulching can reduce the evaporation of soil water, especially in case of drought. Its role in water conservation is particularly critical, as it can effectively improve the water conservation capacity of the soil and promote plant growth. Notably, the ecological membrane can improve the anti-erosion ability of the slope to directly alleviate the problem of erosion. Moreover, it can promote the growth and development of plants; this is conducive to the ecological restoration of the slope and to the realization of a long-term and stable slope protection effect.

**Fig 24:**
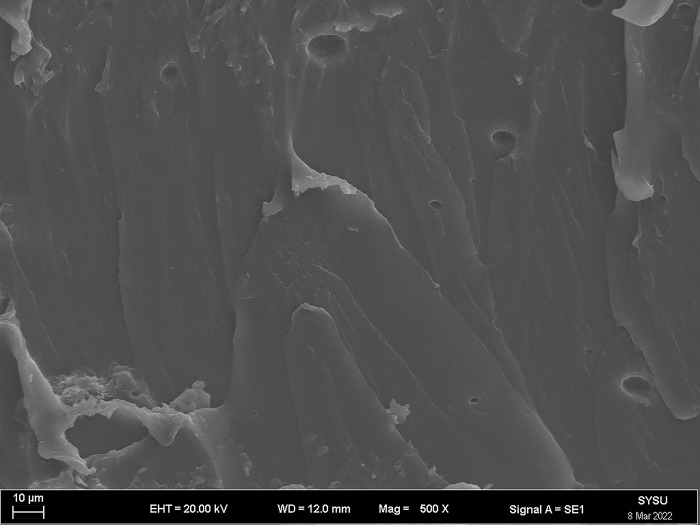
Scanning electron microscopy image of ecological membrane. Ecological membrane sample CK with 0% red bed soil: Magnified 500 times.

**Fig 25:**
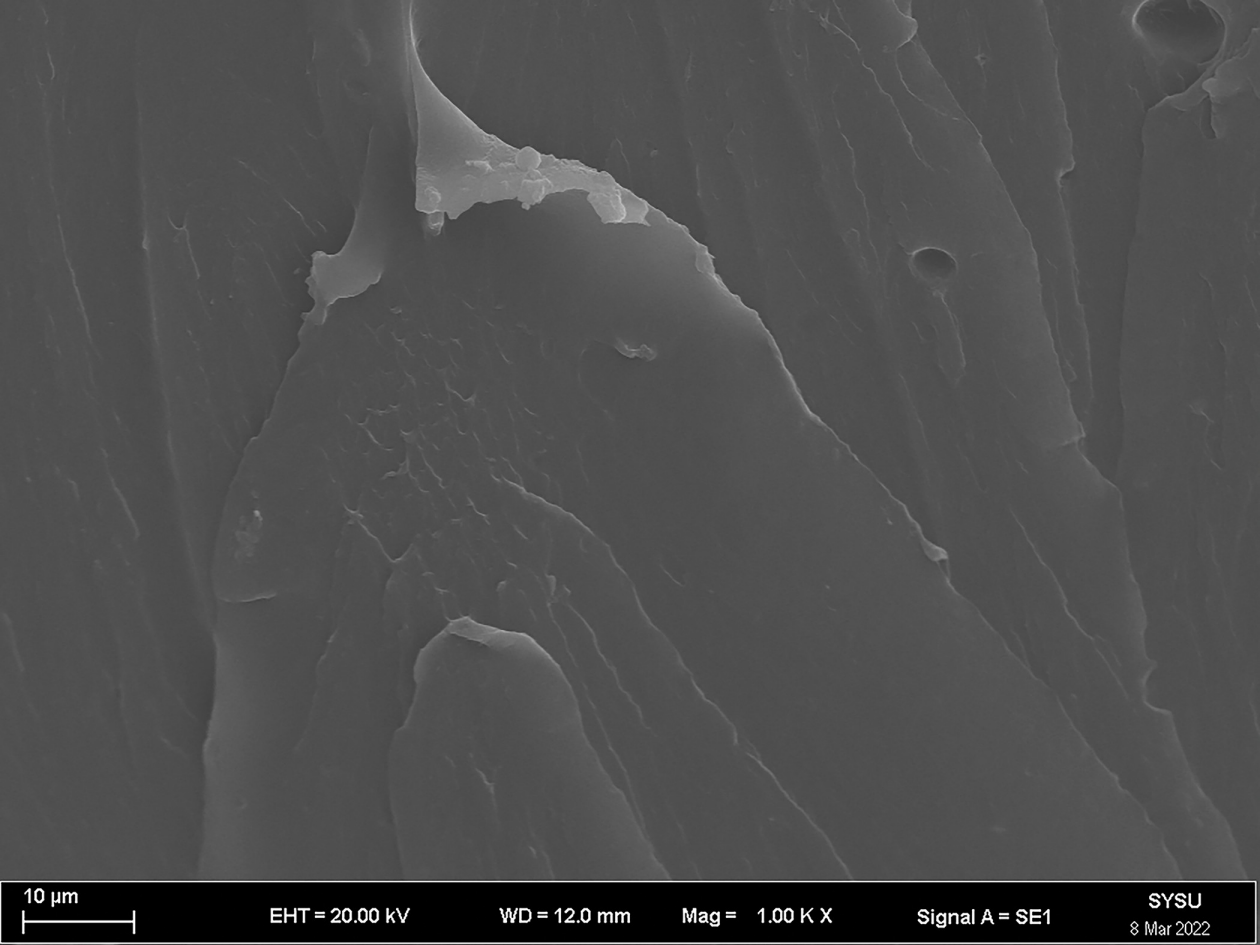
Scanning electron microscopy image of ecological membrane. Ecological membrane sample CK with 0% red bed soil: Magnified 1000 times.

**Fig 26:**
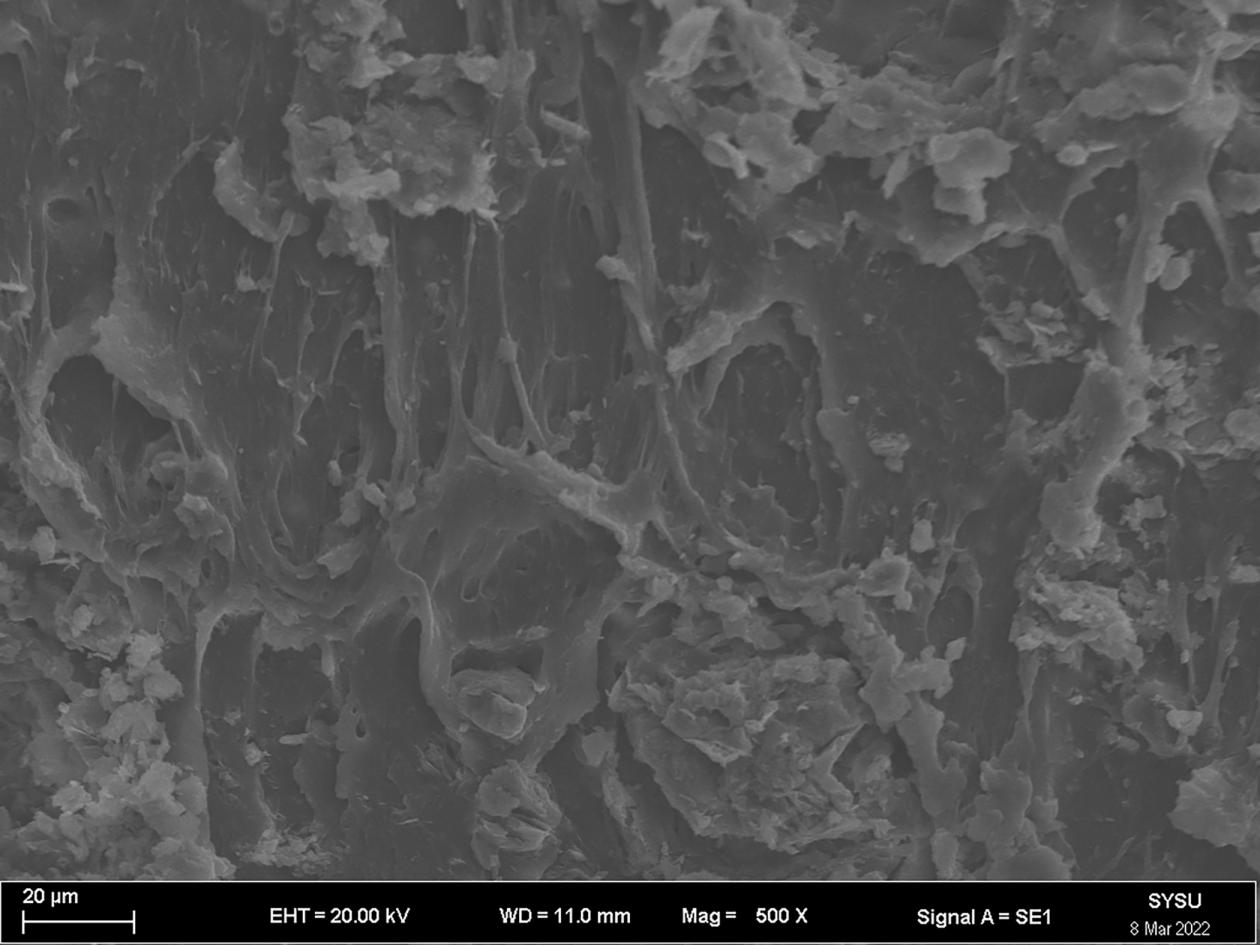
Scanning electron microscopy image of ecological membrane. Ecological membrane sample 3 with 30% red bed soil: Magnified 500 times.

**Fig 27:**
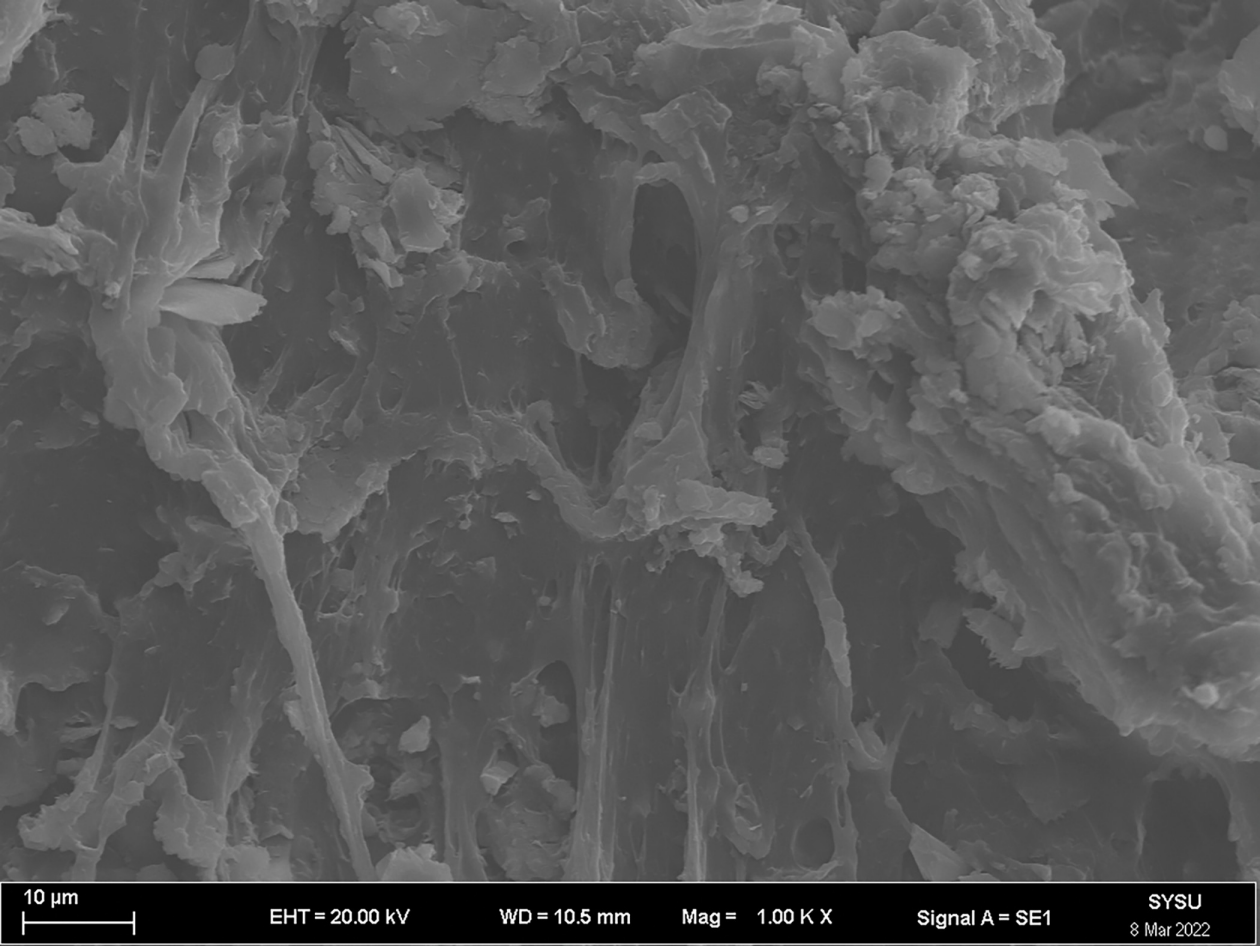
Scanning electron microscopy image of ecological membrane. Ecological membrane sample 3 with 30% red bed soil: Magnified 1000 times.

**Fig 28:**
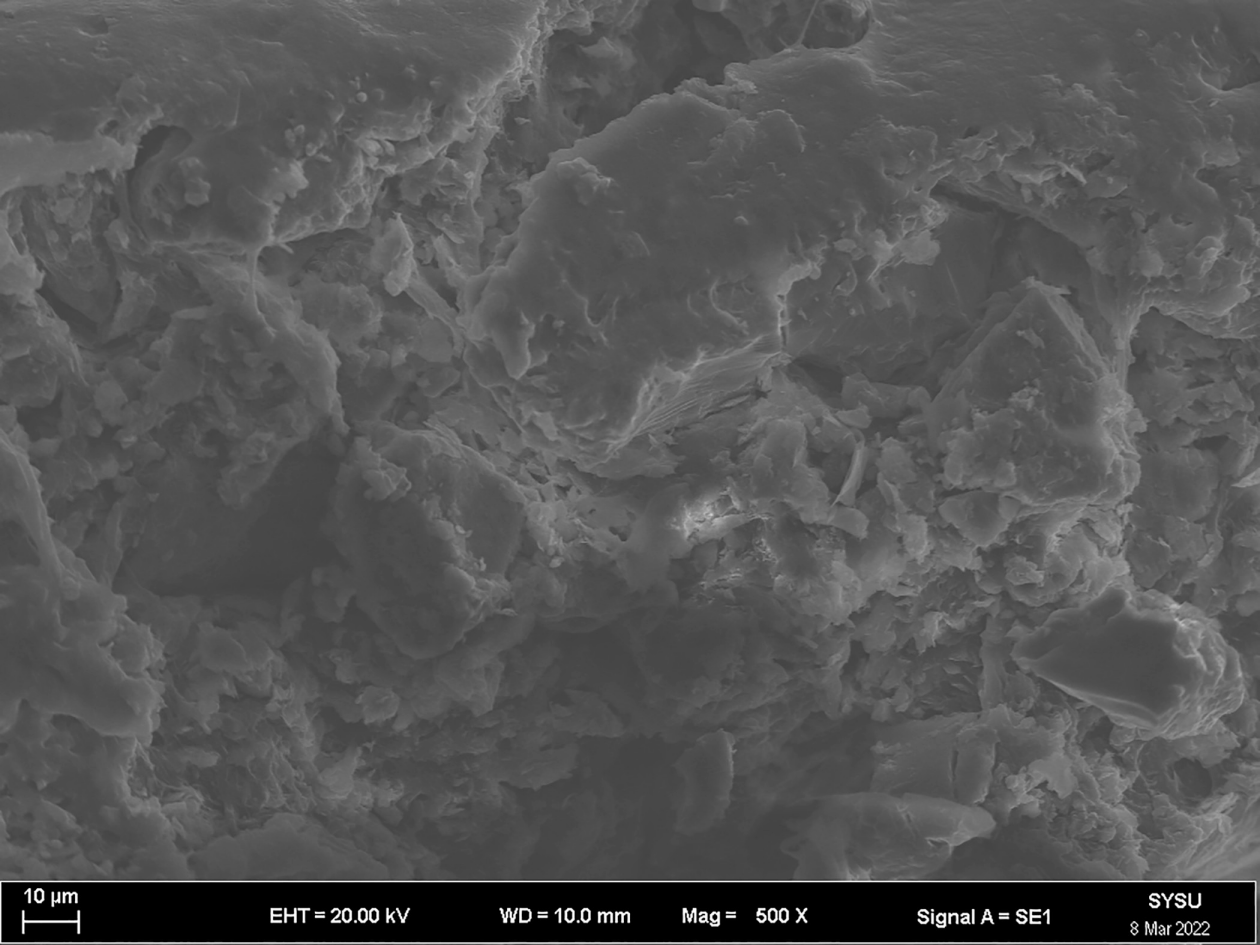
Scanning electron microscopy image of ecological membrane. Ecological membrane sample 5 with 50% red bed soil: Magnified 500 times.

**Fig 29:**
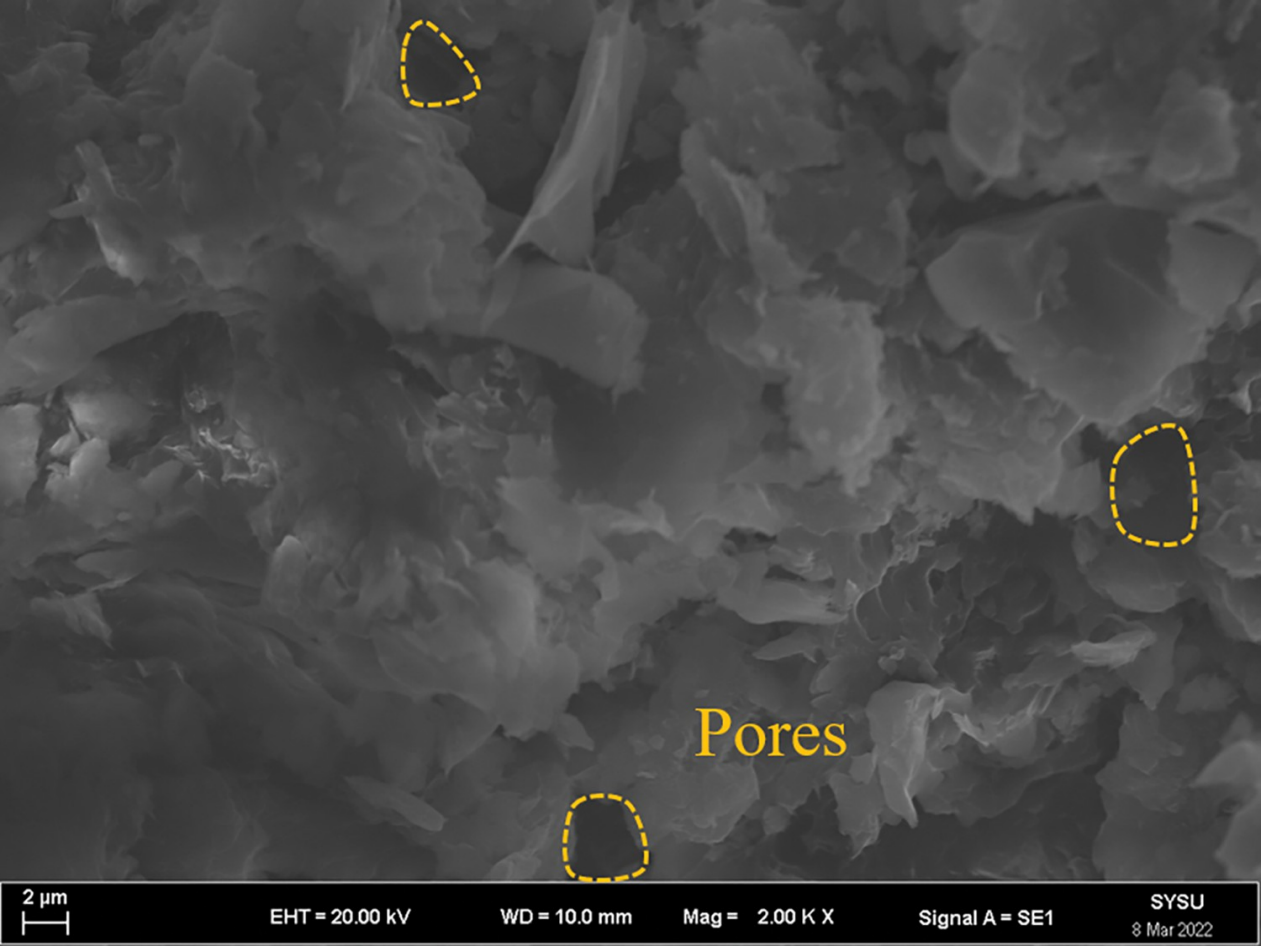
Scanning electron microscopy image of ecological membrane. Ecological membrane sample 5 with 50% red bed soil: Magnified 1000 times.

The strength and viscosity of the ecological membrane materials are affected by the contents of the red bed soil and composite polymer adhesive materials, thus, it can be said that the addition of the materials determines the slope protection performance of the ecological membrane. As shown in Figs 24–29, the micro-structures of the ecological membrane samples under a scanning electron microscope (SEM) show significant differences with respect to different red bed soil contents. This is the fundamental reason for the differences in the performances of the ecological membranes.

It can be seen from the micro-structures of the ecological membrane samples in Figs 24–29 that the sample CK without red bed soil is tight and flat, without an evident particle structure. The SEM image of the ecological membrane with 30% red bed soil shows soil particles arranged in a crisscross manner. The hard red bed soil particles act as the framework of the ecological membrane, forming a closely connected structure under the viscosity of the composite polymer adhesive materials. Therefore, after adding the red bed soil, the strength of the ecological membrane increases, allowing it to better resist the impact of rainfall scouring and protect the soil from erosion. However, the viscosity of the ecological membrane is evidently weakened with the reduction of the adhesive material addition. Figs 28 and 29 shows an SEM image of the ecological membrane with 50% red bed soil; it can be seen that the micro-structural unit of the sample is primarily soil particles, thus, its viscosity is significantly weakened. In its SEM image magnified 1000 times, pores can be seen in some areas. From this, it can be inferred that the addition of the composite polymer adhesive material is not sufficient to form a tight structure, hence, its strength is lesser than that of the sample 3 with 30% red bed soil (as shown in Fig 22). Thus, adding an excessive amount of red bed soil weakens the strength of the ecological membrane and leads to low viscosity, which is not conducive to slope protection.

Based on the addition of red bed soil, this study developed an ecological membrane with strong tensile strength (exceeding 30 N), exhibiting outstanding slope ecological protection performance. To better apply the ecological membrane in soil slope protection, it is necessary to prepare the ecological membrane by using the appropriate proportion of red bed soil and composite polymer adhesive materials. The proportion of the materials determines the strength and viscosity of the ecological membrane. The strength and viscosity of the ecological membrane determine its slope protection performance. It is necessary to develop an ecological membrane with sufficient strength and adequate viscosity corresponding to the actual situation.

## 5 Conclusion

1. The ecological membrane was developed by using green and environmentally friendly composite polymer adhesive materials and red bed soil, which does not produce any substances harmful to the natural environment. Through tensile strength test, viscosity test, anti-erosion test, plant growth test, it can be seen that the ecological membrane comprises a tough and tenacious material with good tensile strength and a certain viscosity, and the ecological membranes have outstanding slope protection performance (including steep slope protection) and the ability to promote the growth of soil plants.
2. The strength of the ecological membrane can be improved by adding an appropriate amount of red bed soil. The ecological membrane with 30% red bed soil has the highest tensile strength. The addition of composite polymer adhesive materials made the ecological membrane elastic–plastic and viscous. The slope protection effect of the ecological membrane is attributed to the combined effect of its own strength and viscosity. The test results showed that the ecological membrane with 20% red bed soil exhibits the strongest anti-erosion performance.
3. In this study, a green and eco-friendly ecological membrane with slope protection and ecological restoration abilities was developed, and its physical and mechanical properties and slope protection performance were studied. This study is still in the laboratory testing stage. We conduct experimental research and share the data. The on-site applicability of this study is actively promoting, and the application research will be presented in another paper.

## Supporting information

S1 File The particle size distribution of the red bed soil. (XLSX)

S2 File Stress–strain curve of membrane. (XLSX)

S3 File Mechanical properties of the ecological membrane. (XLSX)

S4 File Ecological membrane peeling test results. (XLSX)

S5 File Erosion test. (XLSX)

S6 File Analysis of all the test results on the membrane. (XLSX)
